# Supplementary figures and images for: Multimodal HLA-I genotype regulation by human cytomegalovirus US10 and resulting surface patterning
Source: eLife. 2024 Jun 20;13:e85560. doi: 10.7554/eLife.85560 (PMC11189632; doi:10.7554/eLife.85560)

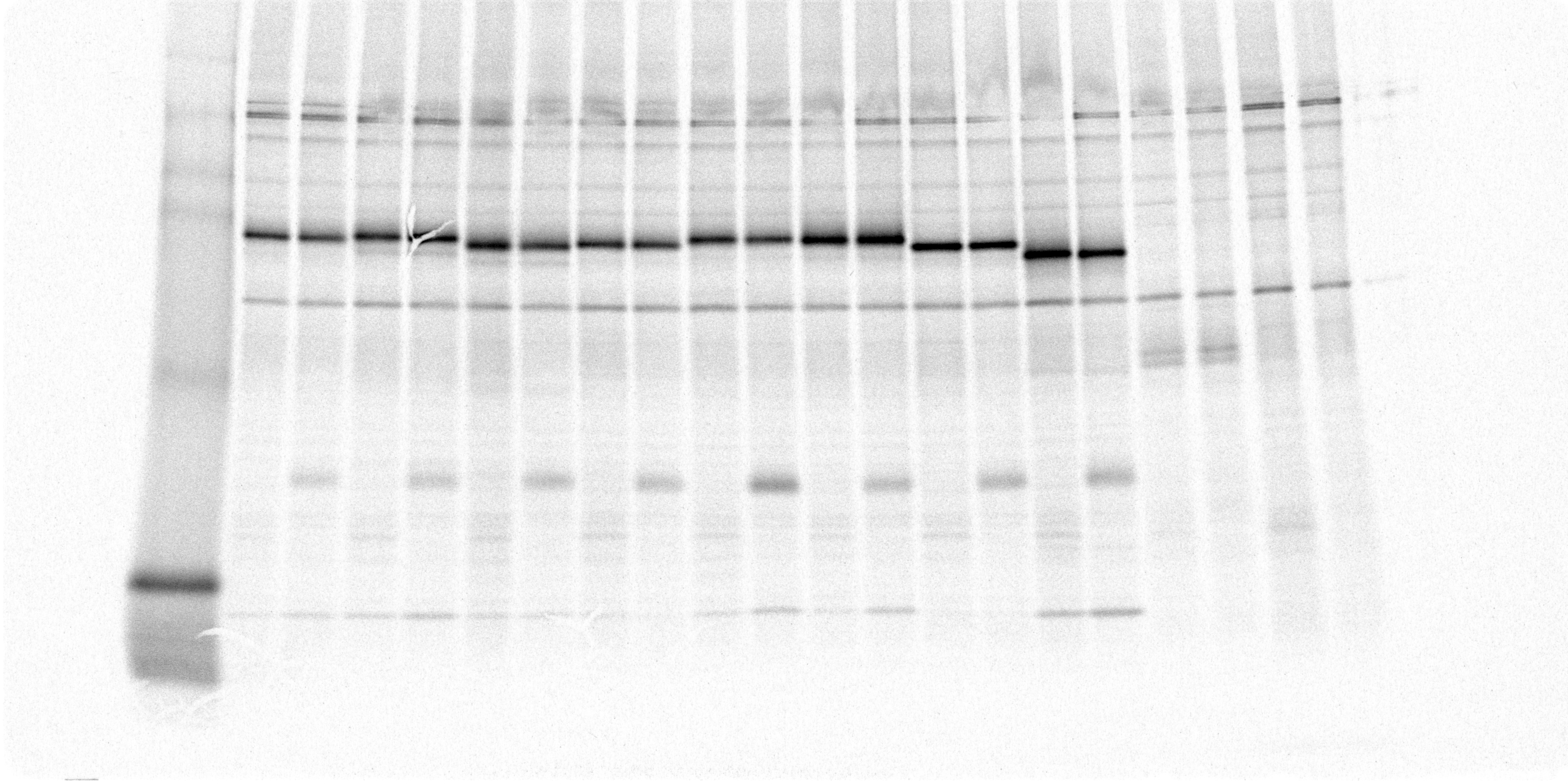

Supplement: Figure 1—source data 1. [file elife-85560-fig1-data1.zip › Figure 1 - Source data 1/Figure 1 - Source data 1_anti-HA_raw data.tif]

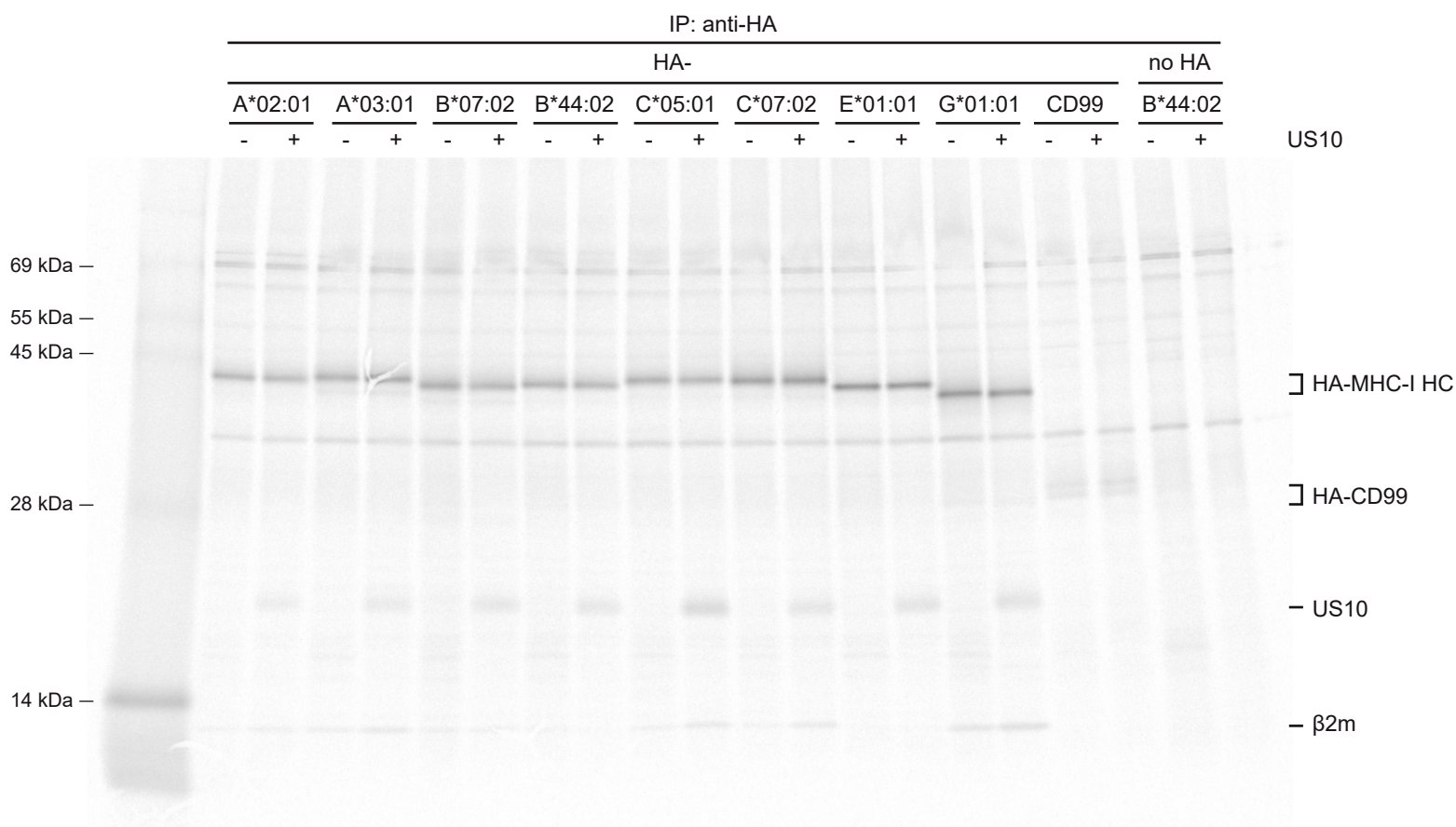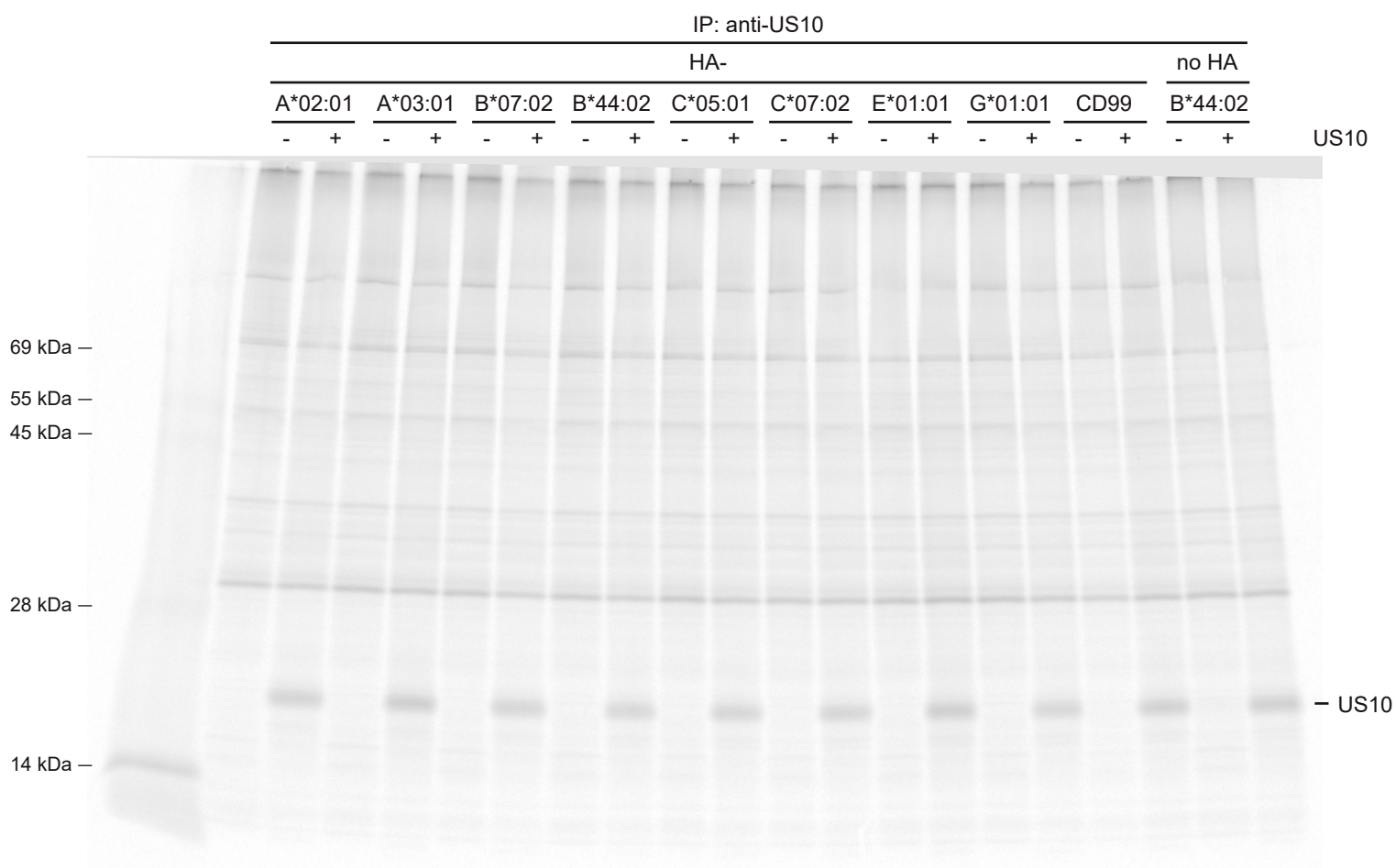

Figure 1C: uncropped gels

Supplement: Figure 1—source data 1. [file elife-85560-fig1-data1.zip › Figure 1 - Source data 1/Figure_1C_uncropped.pdf]

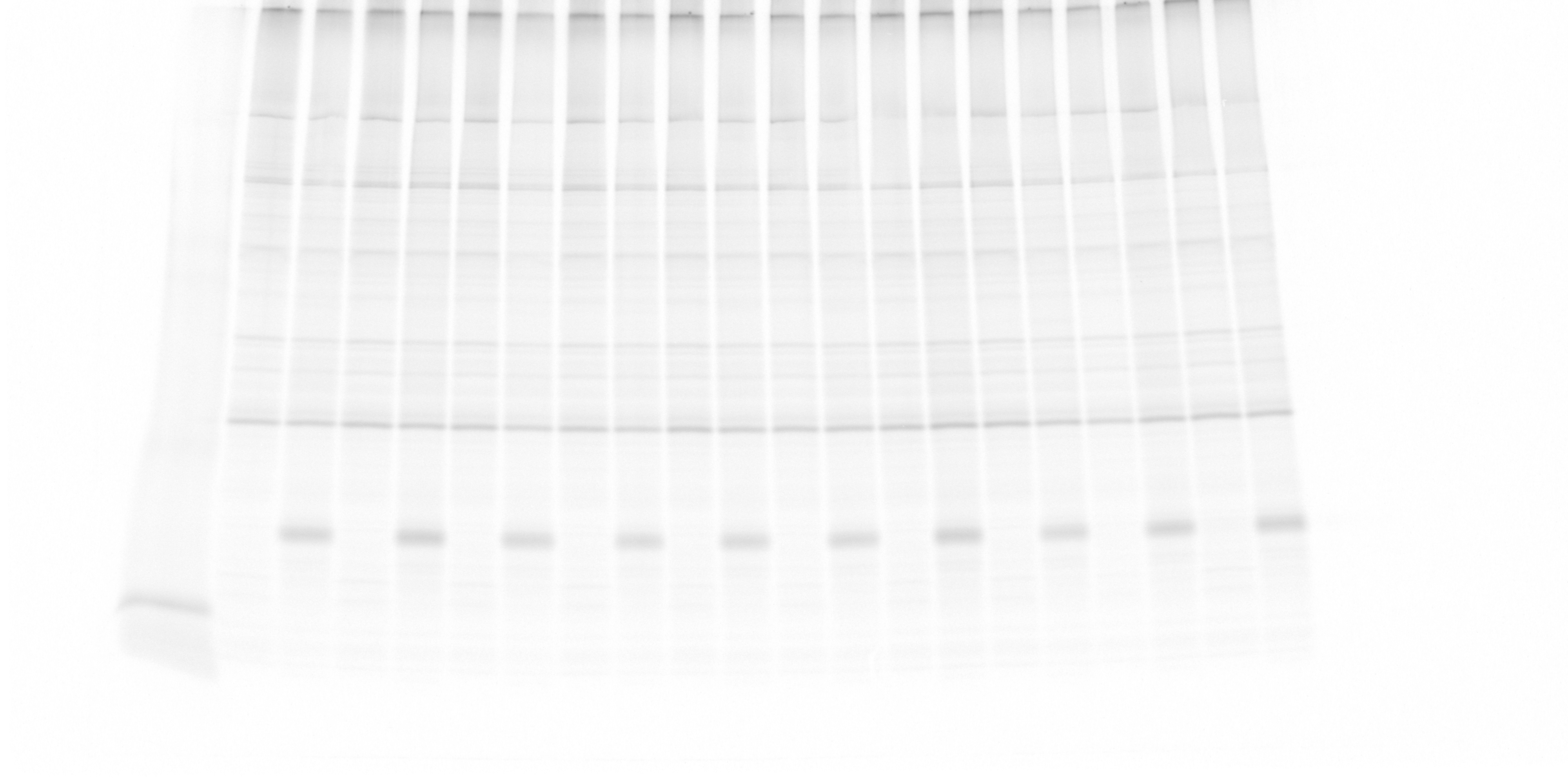

Supplement: Figure 1—source data 1. [file elife-85560-fig1-data1.zip › Figure 1 - Source data 1/Figure 1 - Source data 1_anti-US10_raw data.tif]

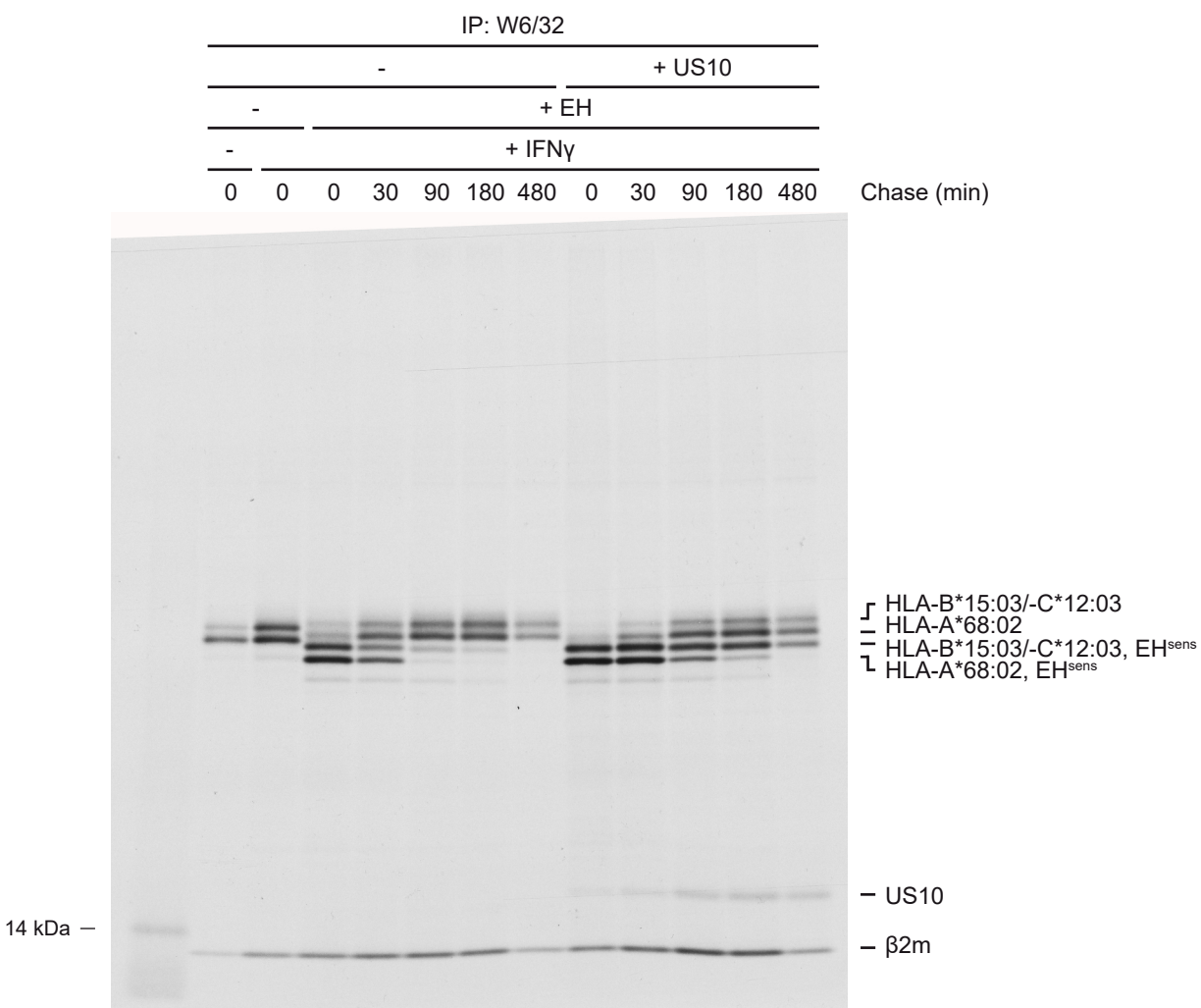

Figure 2A: uncropped gel

Supplement: Figure 2—source data 1. [file elife-85560-fig2-data1.zip › Figure 2 - Source data 1/Figure_2A_uncropped.pdf]

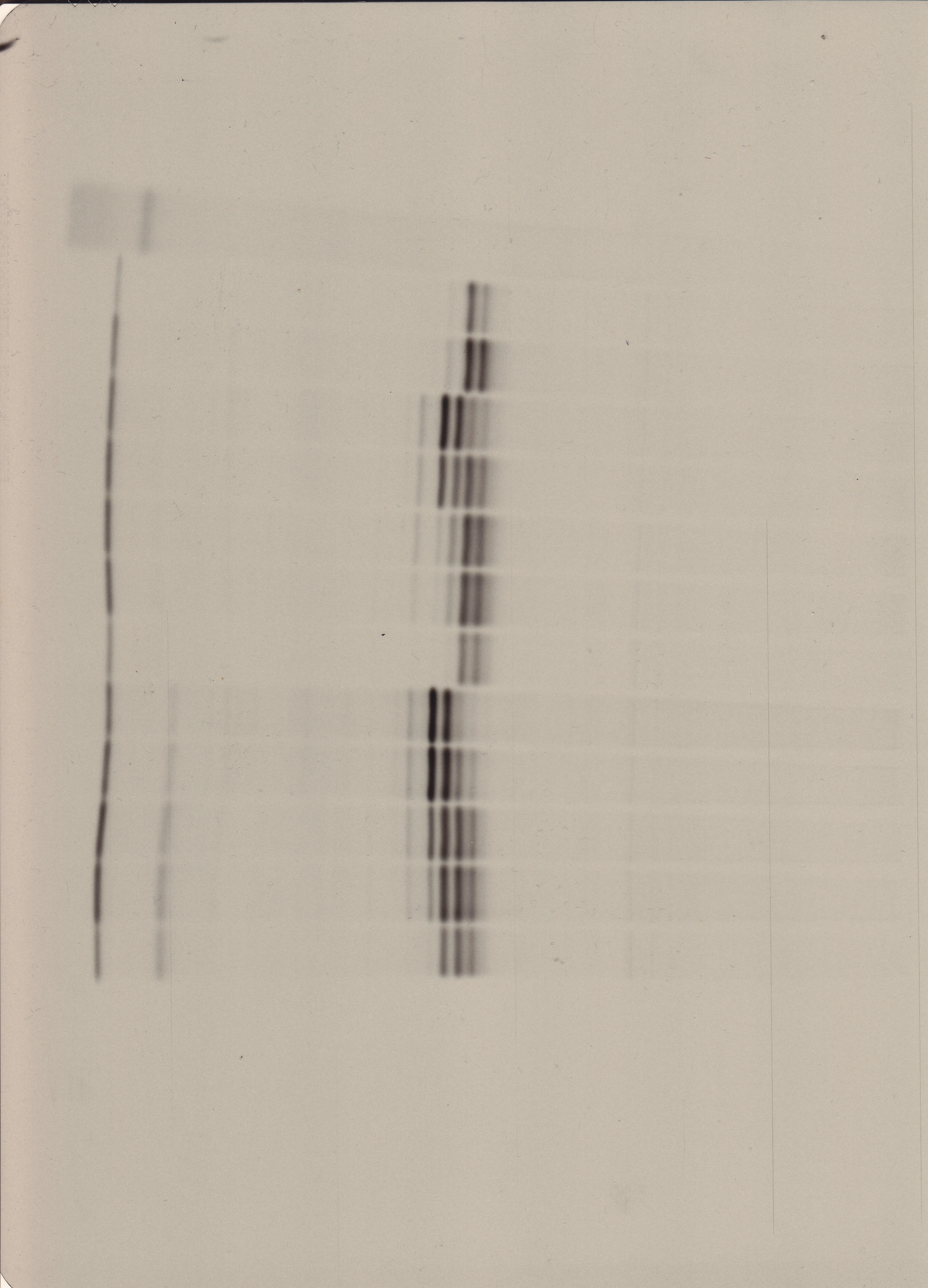

Supplement: Figure 2—source data 1. [file elife-85560-fig2-data1.zip › Figure 2 - Source data 1/Figure 2 - Source data 1_raw data.tif]

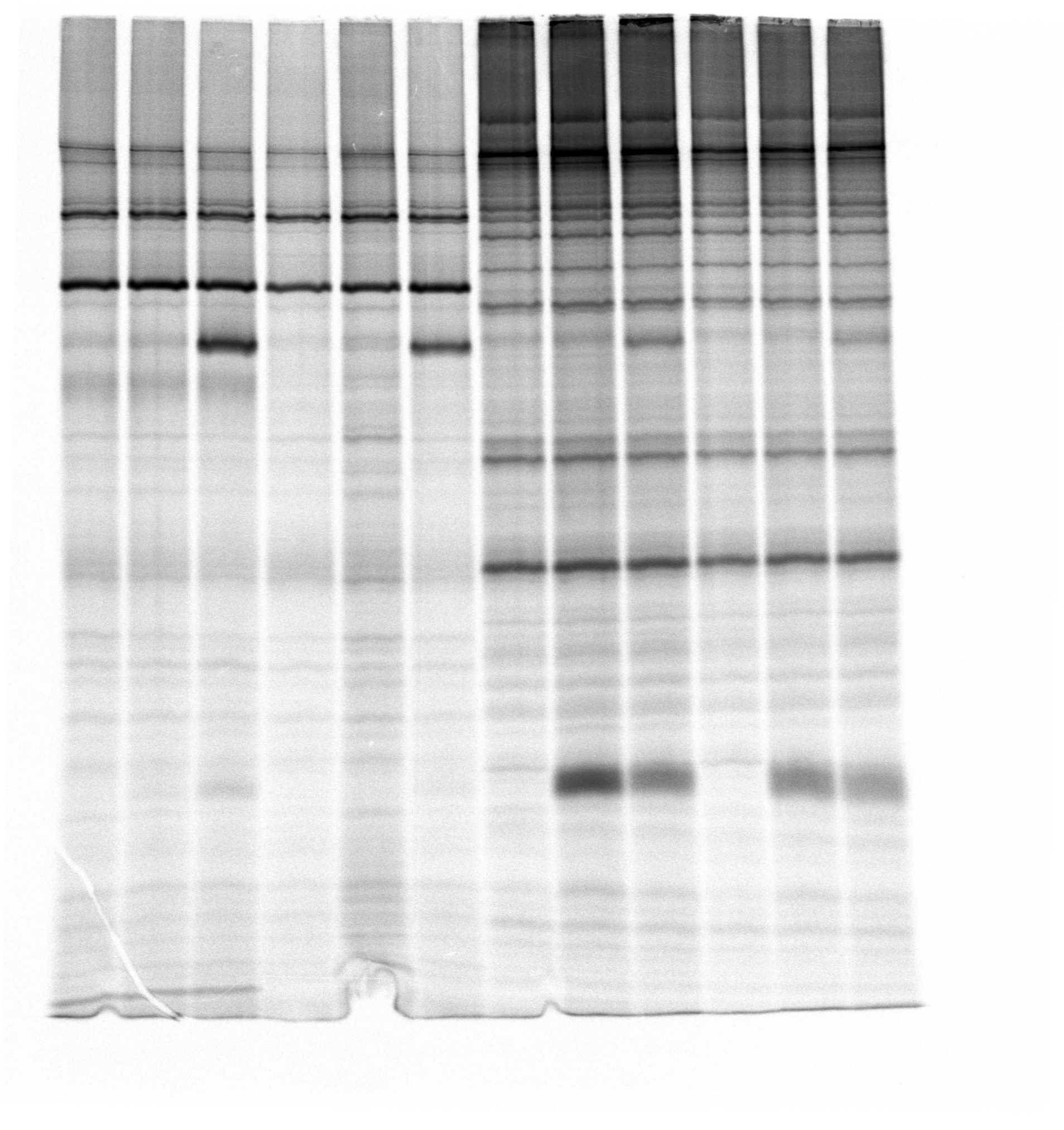

Supplement: Figure 2—source data 2. [file elife-85560-fig2-data2.zip › Figure 2 - Source data 2/Figure 2 - Source data 3_raw data.tif]

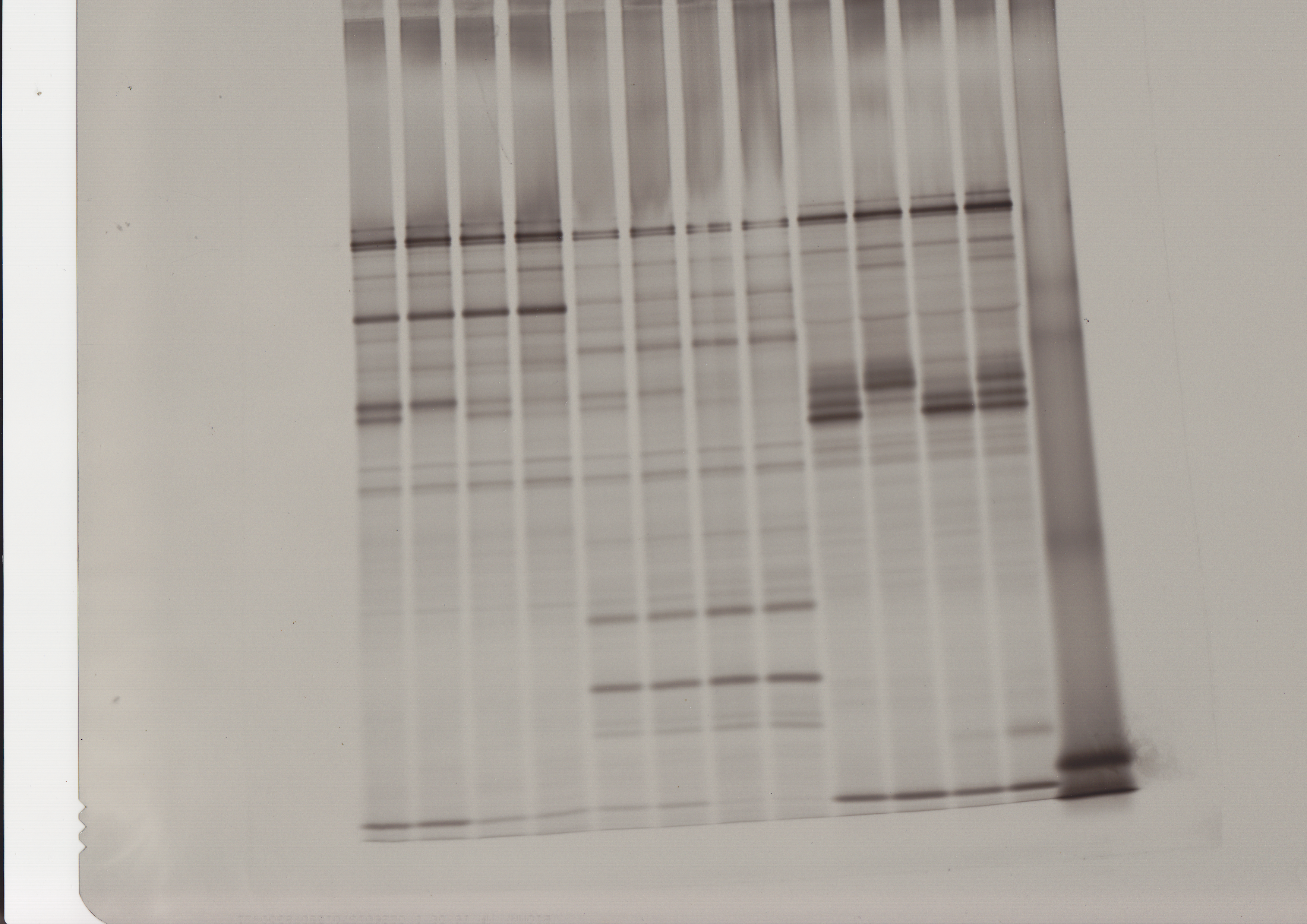

Supplement: Figure 2—source data 2. [file elife-85560-fig2-data2.zip › Figure 2 - Source data 2/Figure 2 - Source data 2_raw data.tif]

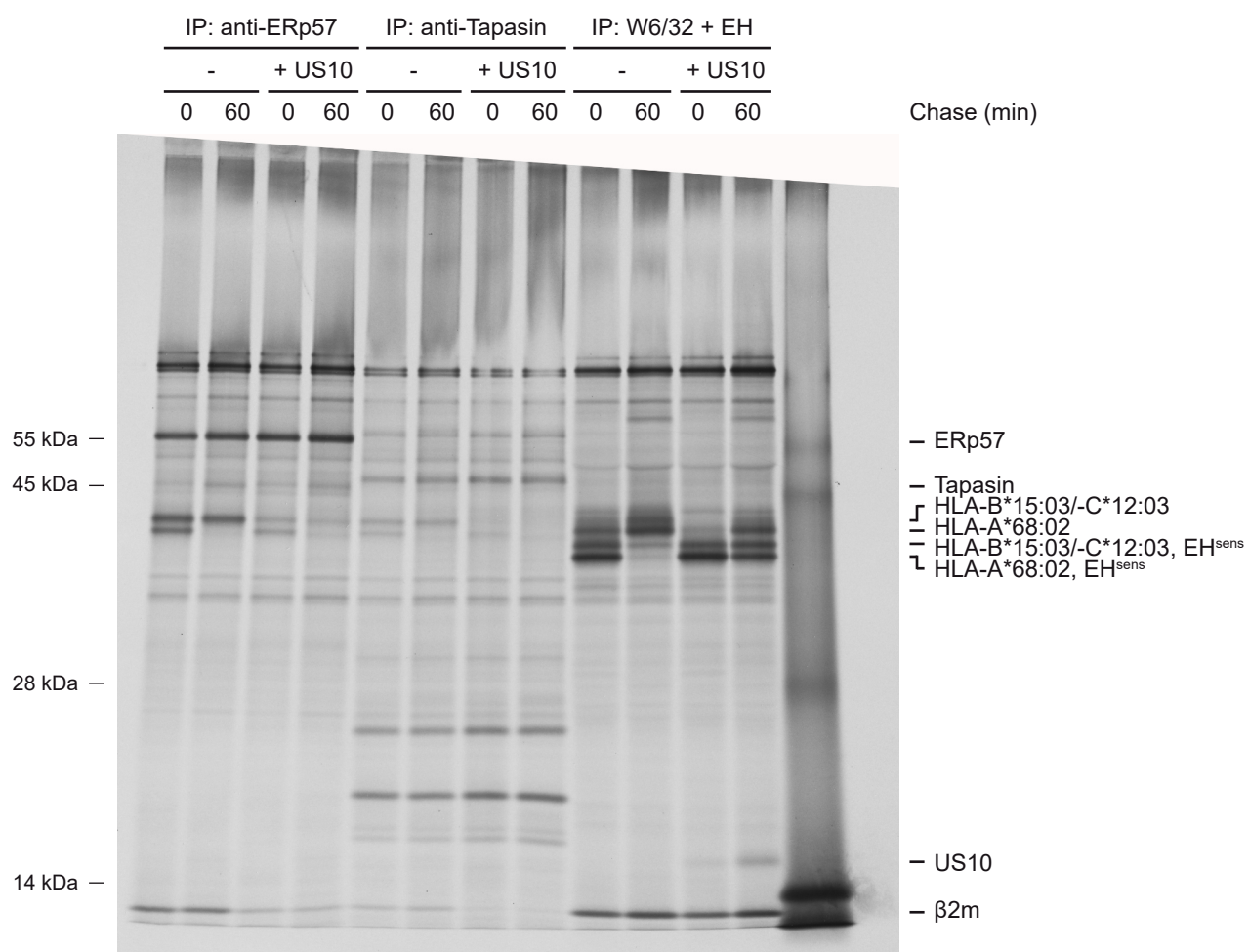

**Figure 2C: uncropped gel**

Supplement: Figure 2—source data 2. [file elife-85560-fig2-data2.zip › Figure 2 - Source data 2/Figure_2C_uncropped.pdf]

IP: anti-ERp57

IP: anti-US10

|   |   |   |   |
|---|---|---|---|
| - | + | + | + |
| + | + | + | - |
| - | - | + | + |

|   |   |   |   |         |
|---|---|---|---|---------|
| - | + | + | + | US10    |
| + | + | + | - | HLA-I   |
| - | - | + | + | Tapasin |

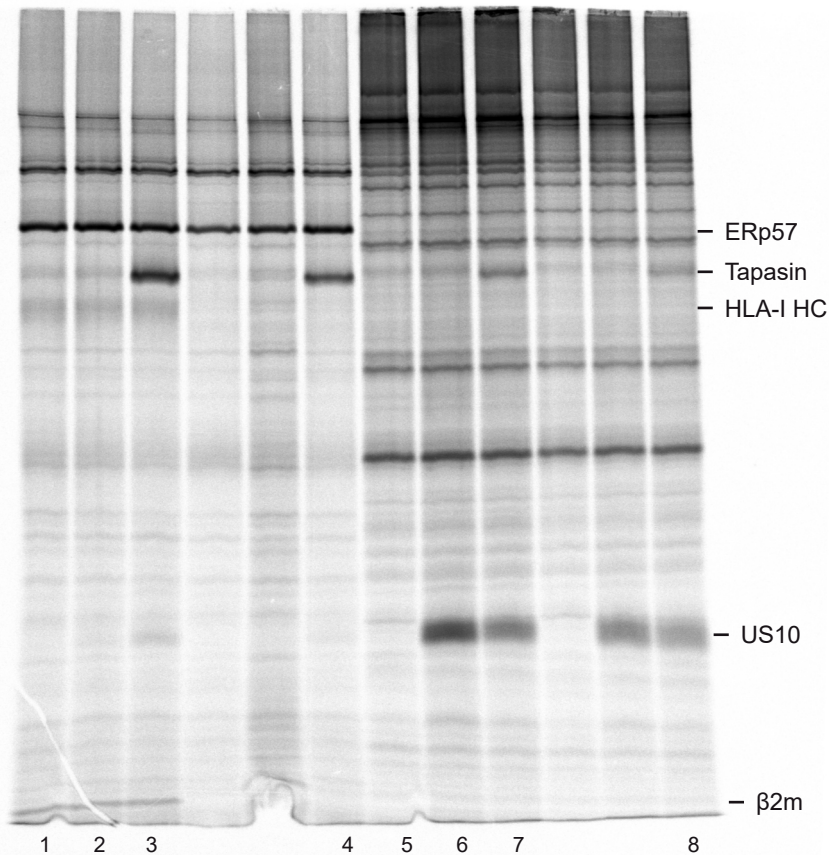

Supplement: Figure 2—source data 3. [file elife-85560-fig2-data3.zip › Figure 2 - Source data 3/Figure_2E_uncropped.pdf]

IP:  
W6/32

WT  
HLA-I KO

HLA-I HC

—  $\beta$ 2m

55 kDa —

45 kDa —

28 kDa —

14 kDa —

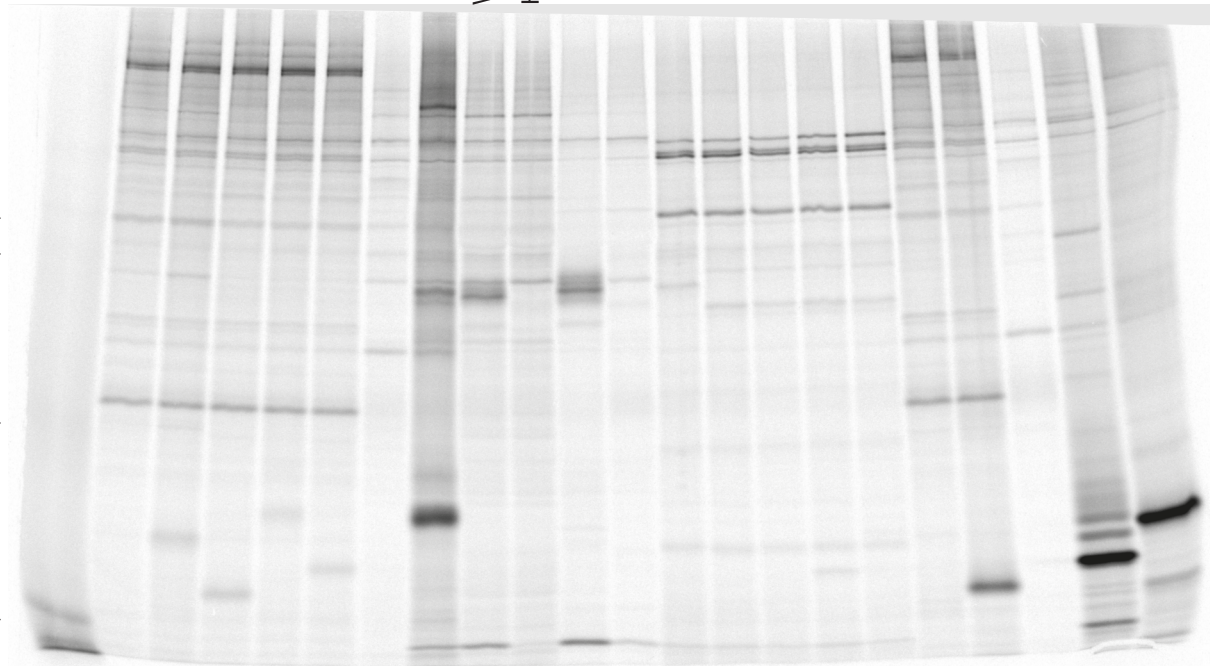

Supplement: Figure 2—figure supplement 1—source data 1. [file elife-85560-fig2-figsupp1-data1.zip › Figure 2 - Figure supplement 1 - Source data 1/Suppl_figure_1C_uncropped.pdf]

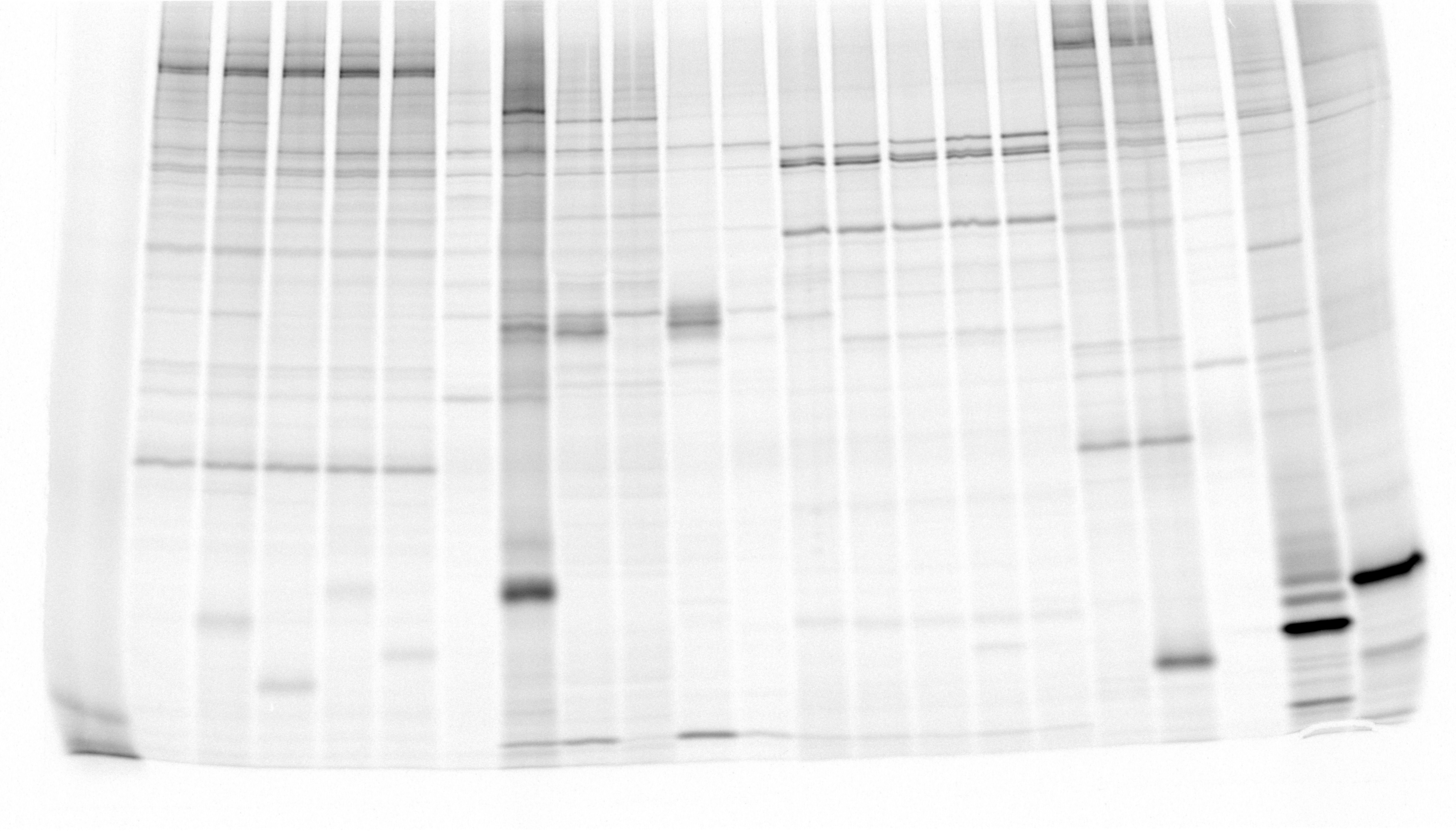

Supplement: Figure 2—figure supplement 1—source data 1. [file elife-85560-fig2-figsupp1-data1.zip › Figure 2 - Figure supplement 1 - Source data 1/Figure 2 - Figure supplement 1 - Source data 1_raw data.tif]

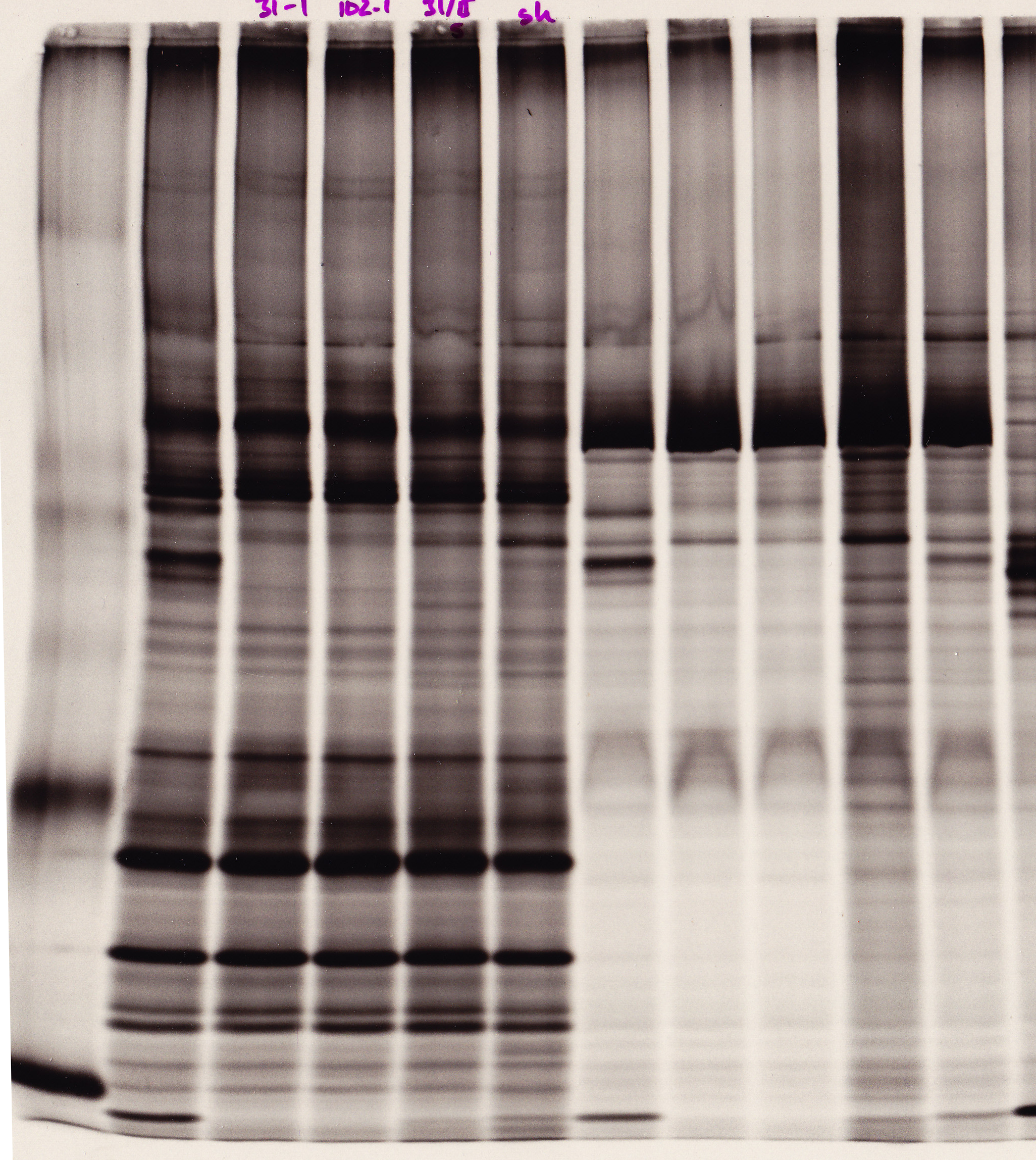

Supplement: Figure 3—figure supplement 1—source data 1. [file elife-85560-fig3-figsupp1-data1.zip › Figure 3 - Figure supplement 1 - Source data 1/Figure 3 - Figure supplement 1 - Source data 1_long_raw data.tif]

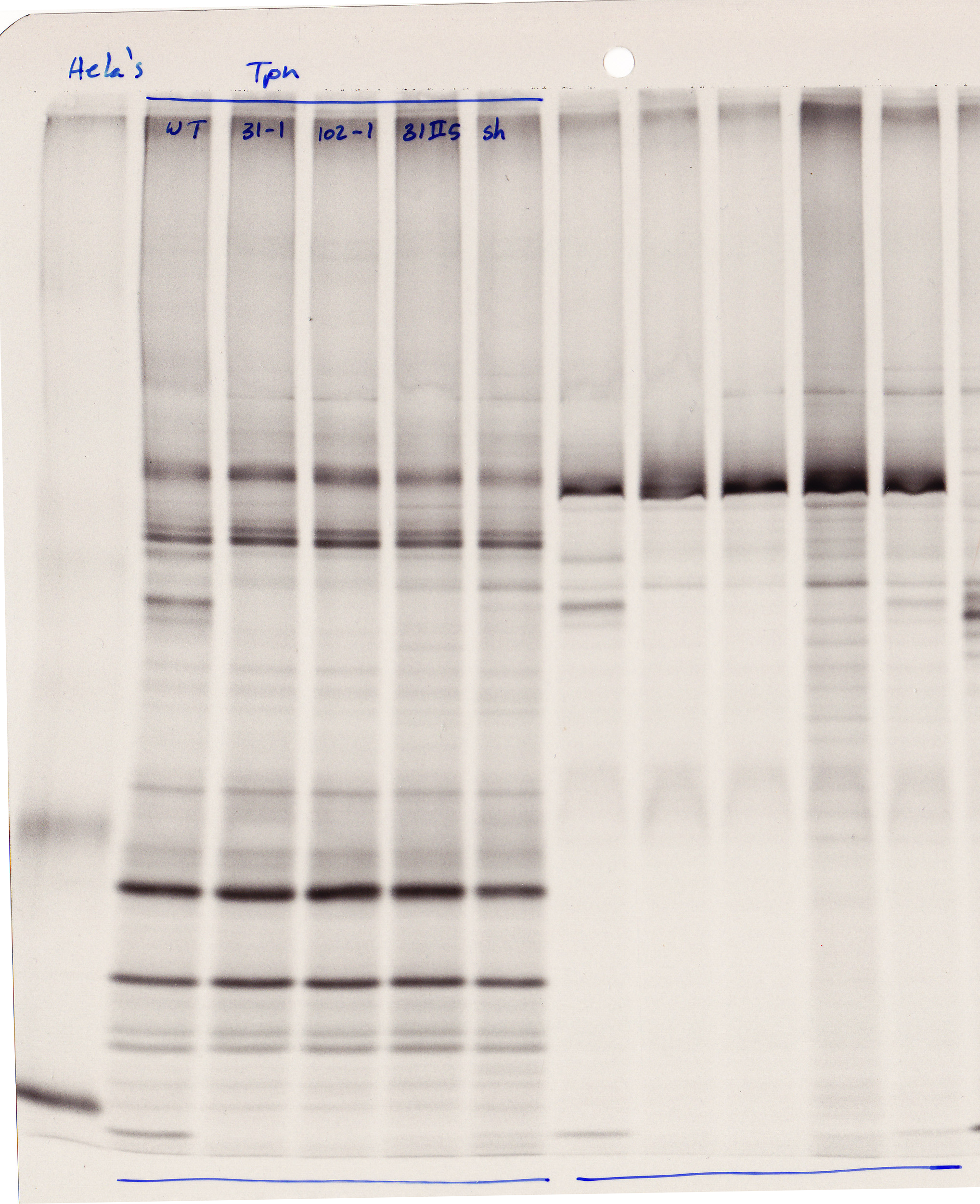

Supplement: Figure 3—figure supplement 1—source data 1. [file elife-85560-fig3-figsupp1-data1.zip › Figure 3 - Figure supplement 1 - Source data 1/Figure 3 - Figure supplement 1 - Source data 1_short_raw data.tif]

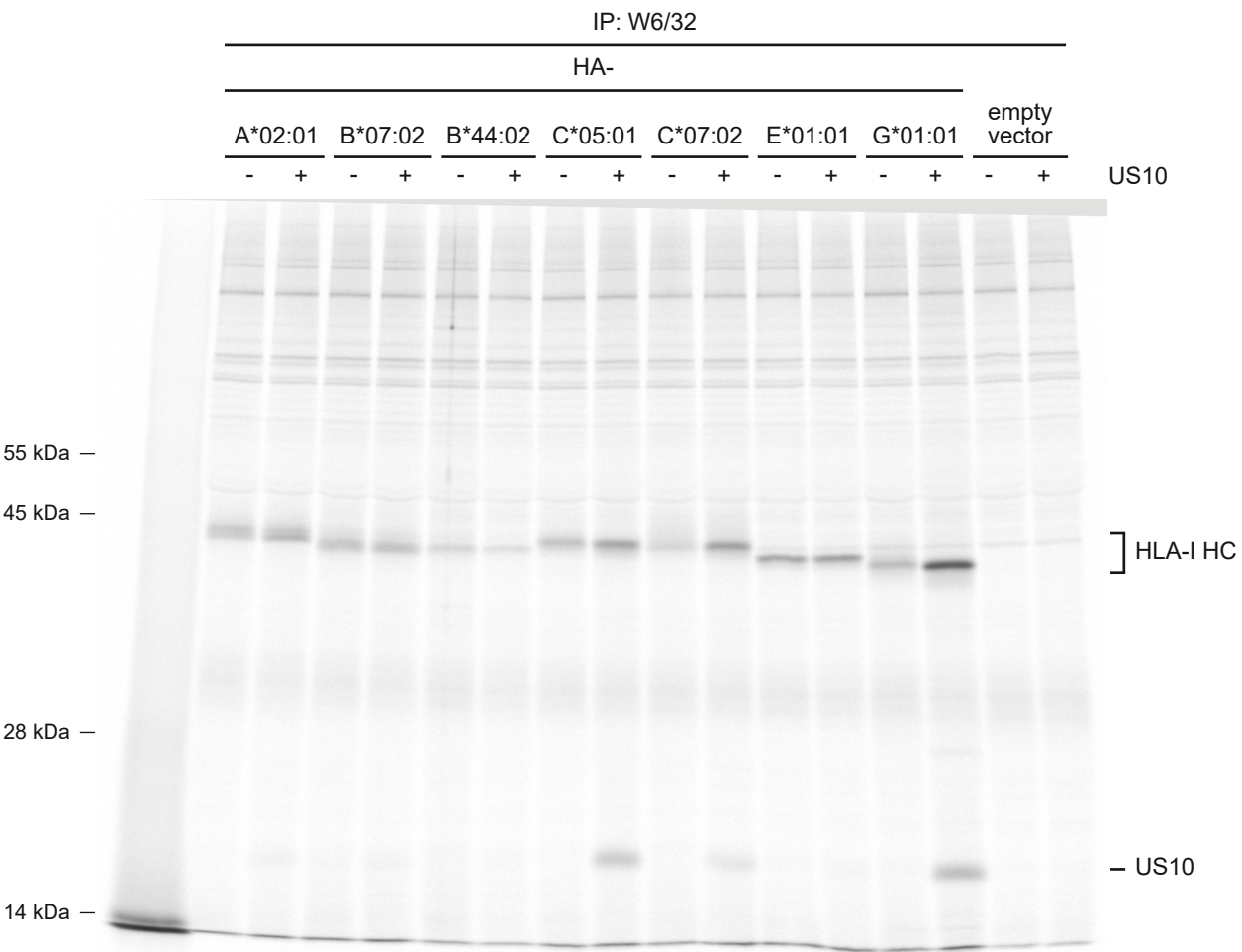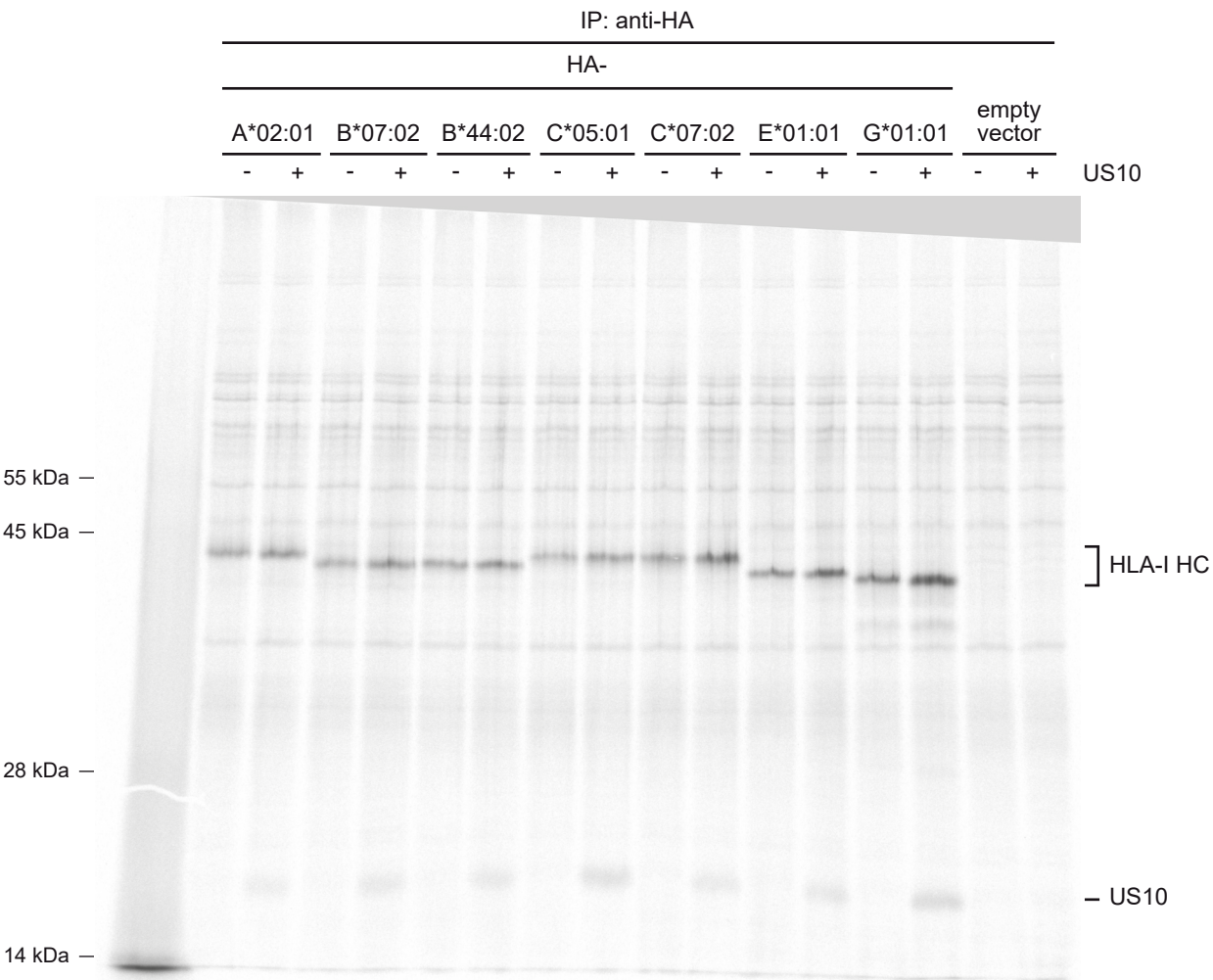

Figure 4A: uncropped gels (1)

Supplement: Figure 4—source data 1. [file elife-85560-fig4-data1.zip › Figure 4 - Source data 1/Figure_4A_uncropped-1.pdf]

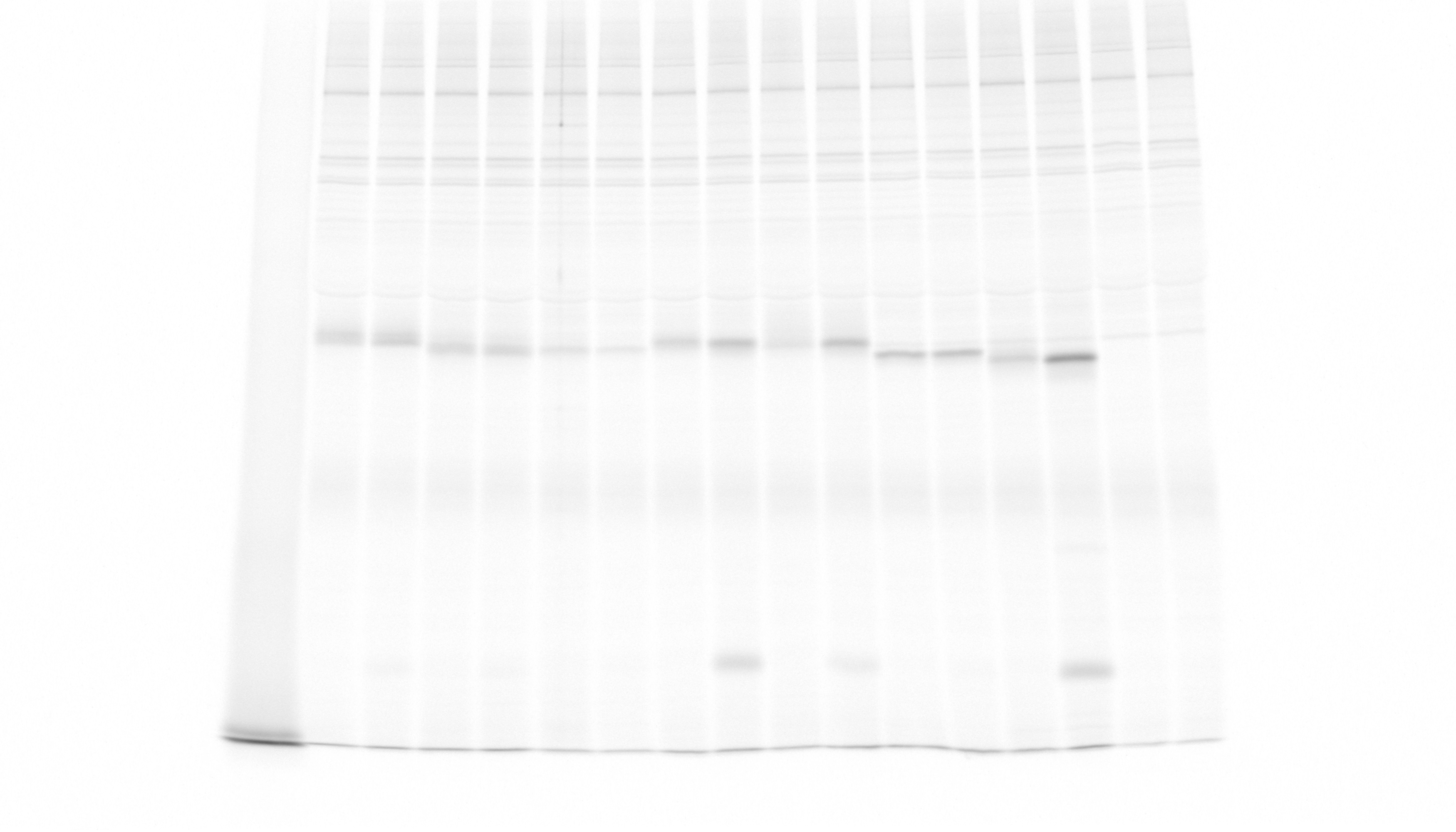

Supplement: Figure 4—source data 1. [file elife-85560-fig4-data1.zip › Figure 4 - Source data 1/Figure 4 - Source data 1_W632_raw data.tif]

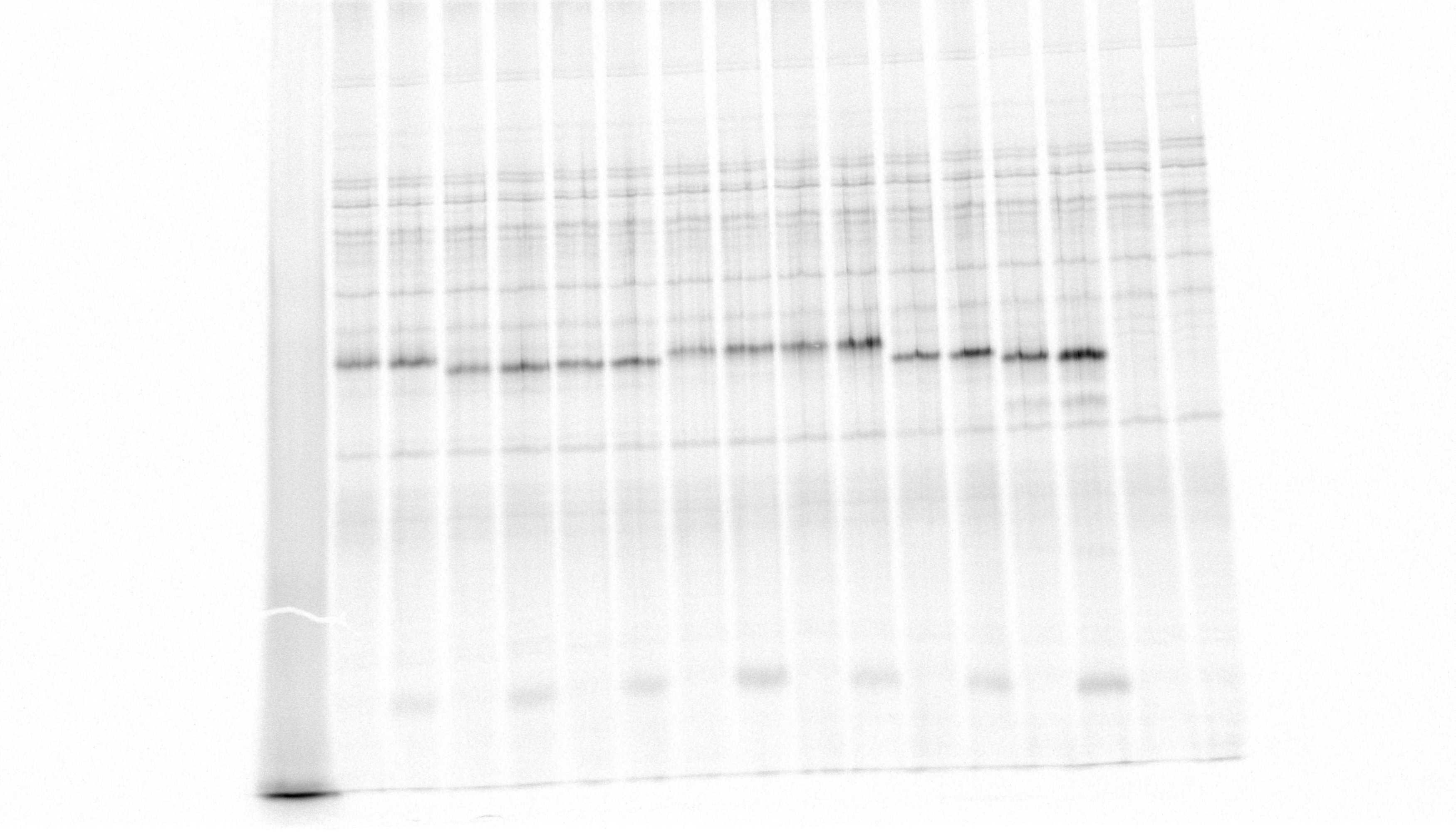

Supplement: Figure 4—source data 1. [file elife-85560-fig4-data1.zip › Figure 4 - Source data 1/Figure 4 - Source data 1_anti-HA_raw data.tif]

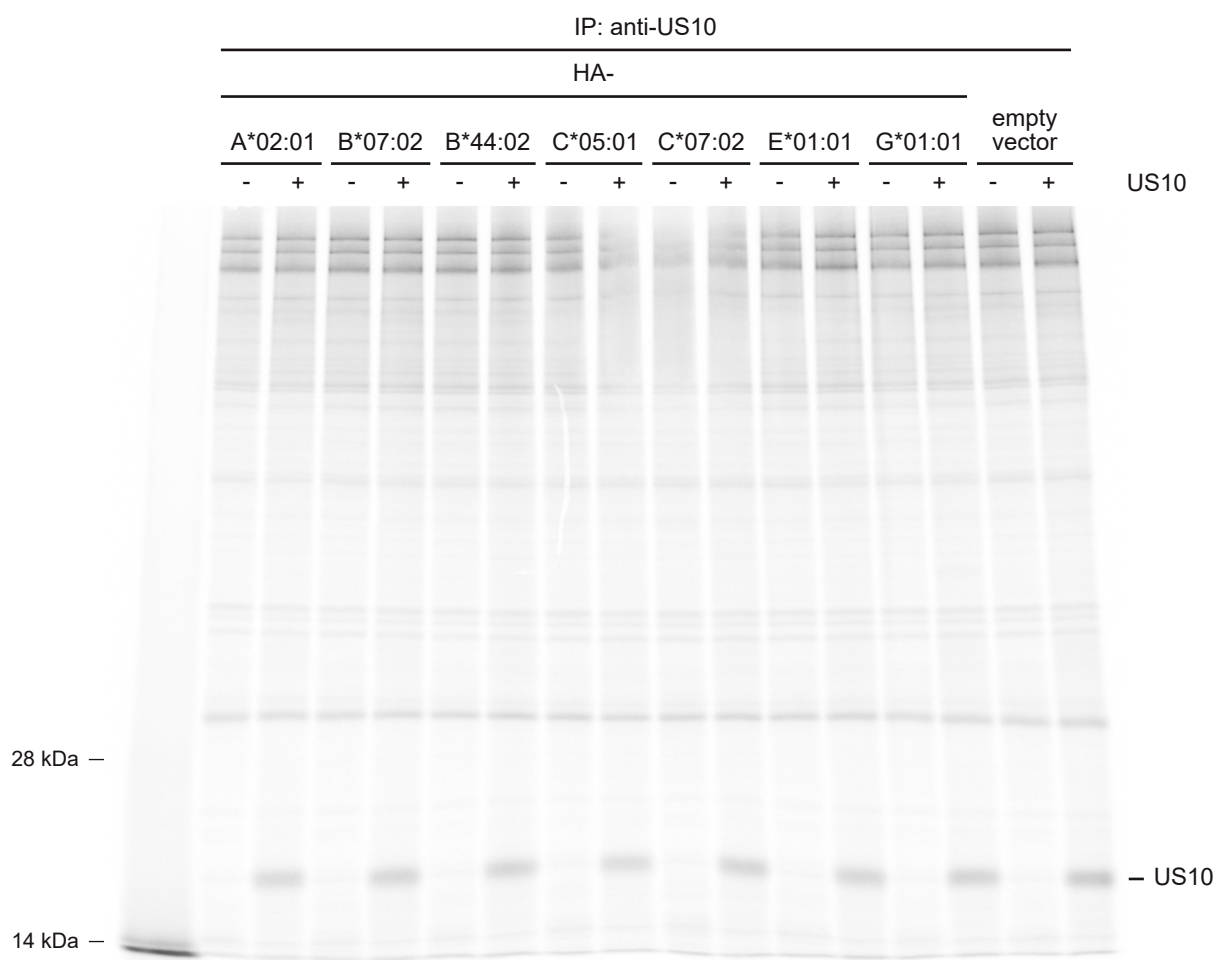

Figure 4A: uncropped gels (2)

Supplement: Figure 4—source data 2. [file elife-85560-fig4-data2.zip › Figure 4 - Source data 2/Figure_4A_uncropped-2.pdf]

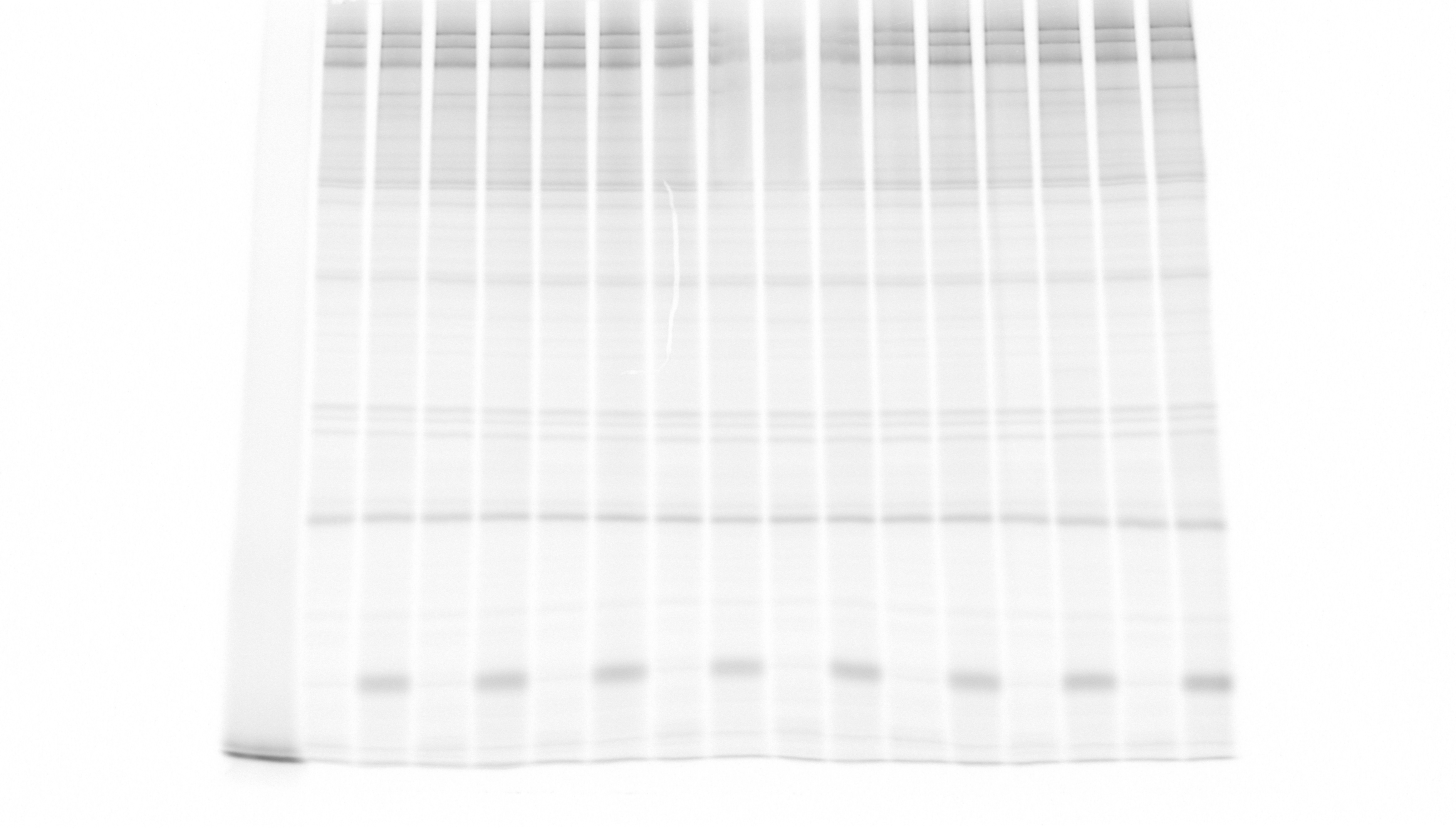

Supplement: Figure 4—source data 2. [file elife-85560-fig4-data2.zip › Figure 4 - Source data 2/Figure 4 - Source data 2_anti-US10_raw data.tif]

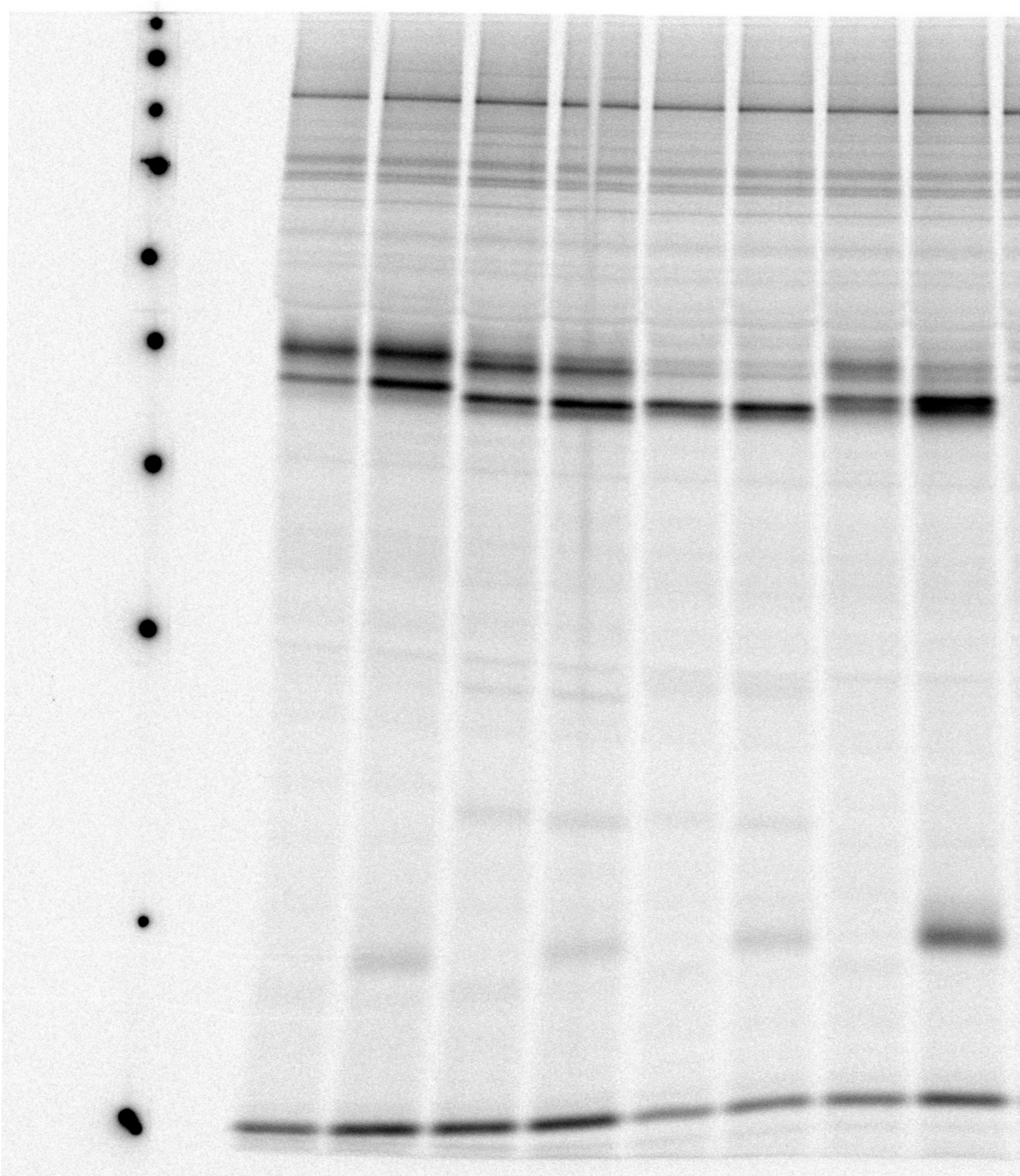

Supplement: Figure 4—source data 3. [file elife-85560-fig4-data3.zip › Figure 4 - Source data 3/Figure 4 - Source data 3_raw data.tif]

IP: W6/32

~A\*02:01    ~B\*07:02    ~B\*44:02    ~C\*05:01

-    +    -    +    -    +    -    +    US10

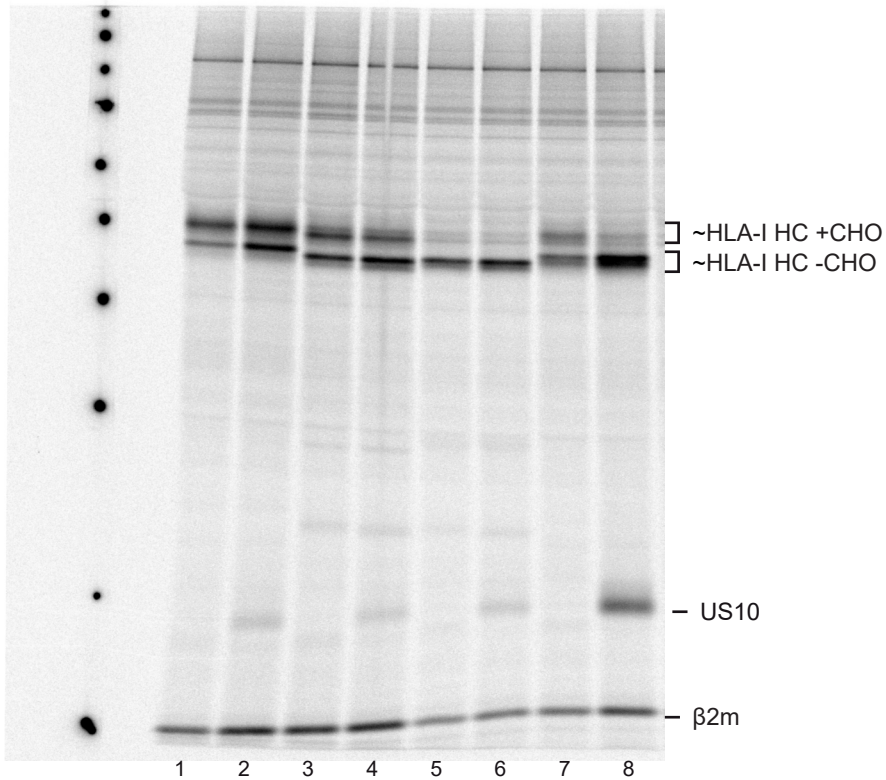

Supplement: Figure 4—source data 3. [file elife-85560-fig4-data3.zip › Figure 4 - Source data 3/Figure_4C_uncropped.pdf]

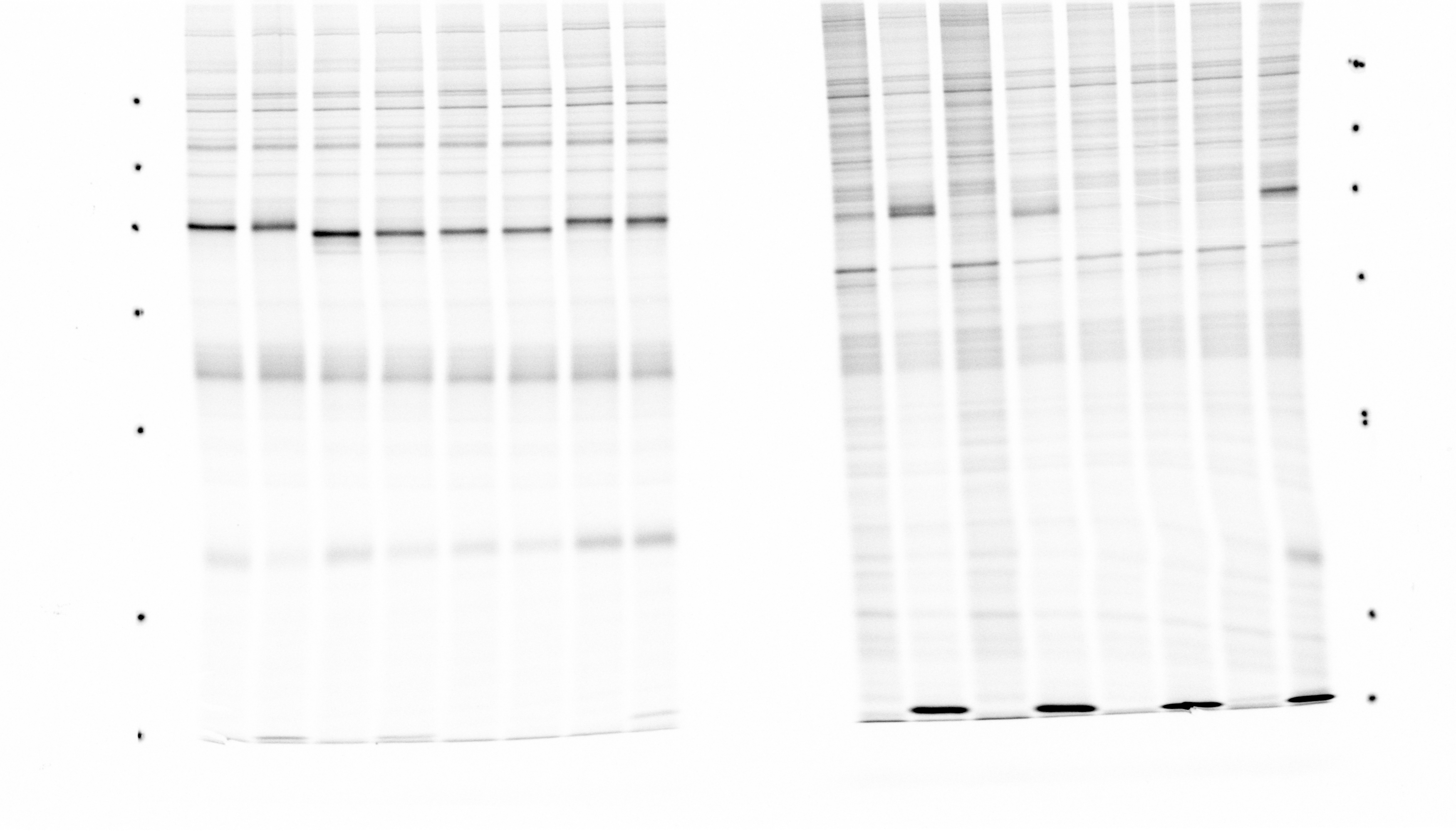

Supplement: Figure 4—source data 4. [file elife-85560-fig4-data4.zip › Figure 4 - Source data 4/Figure 4 - Source data 4_anti-HA_BBM1_raw data.tif]

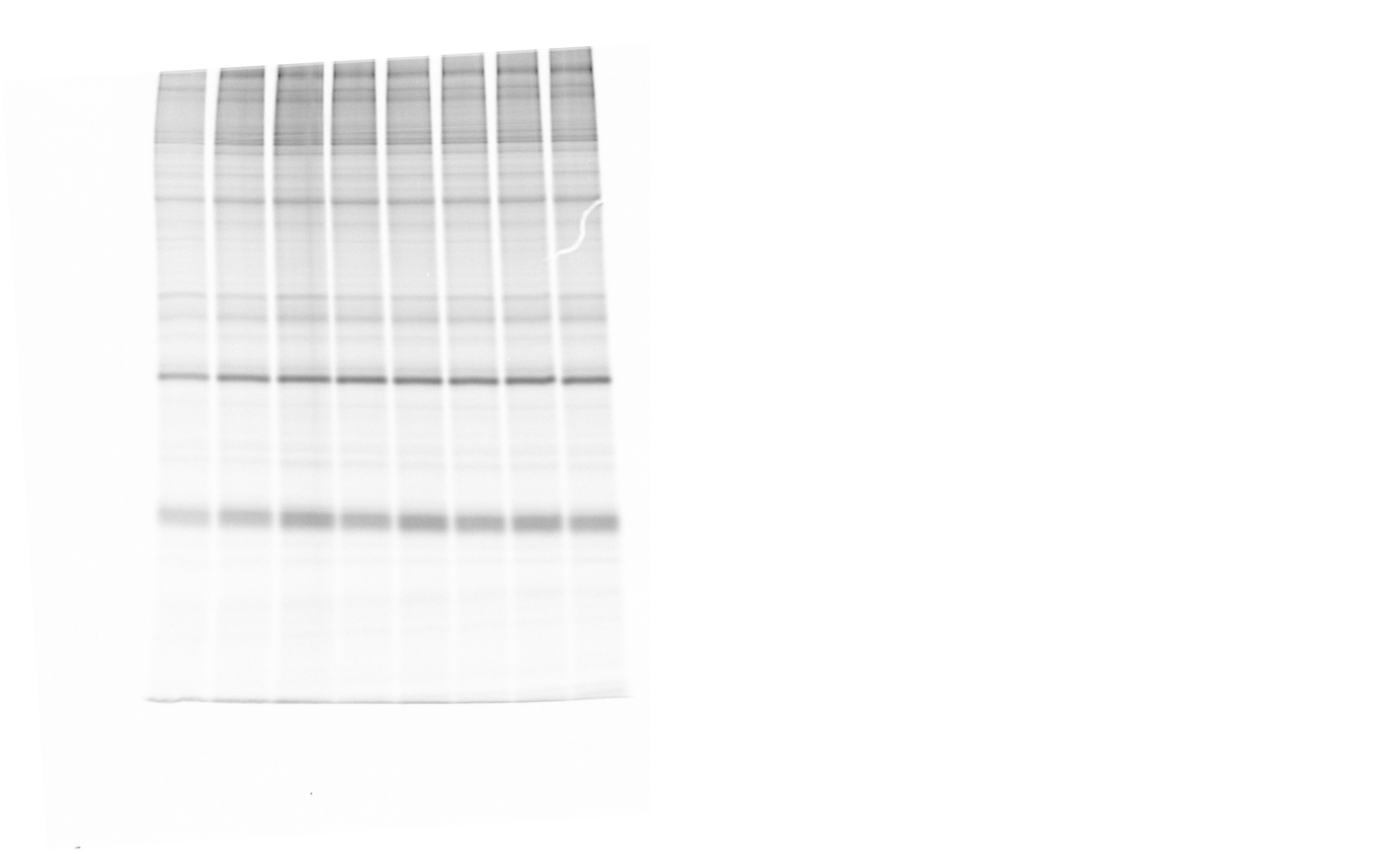

Supplement: Figure 4—source data 4. [file elife-85560-fig4-data4.zip › Figure 4 - Source data 4/Figure 4 - Source data 4_anti-US10_raw data.tif]

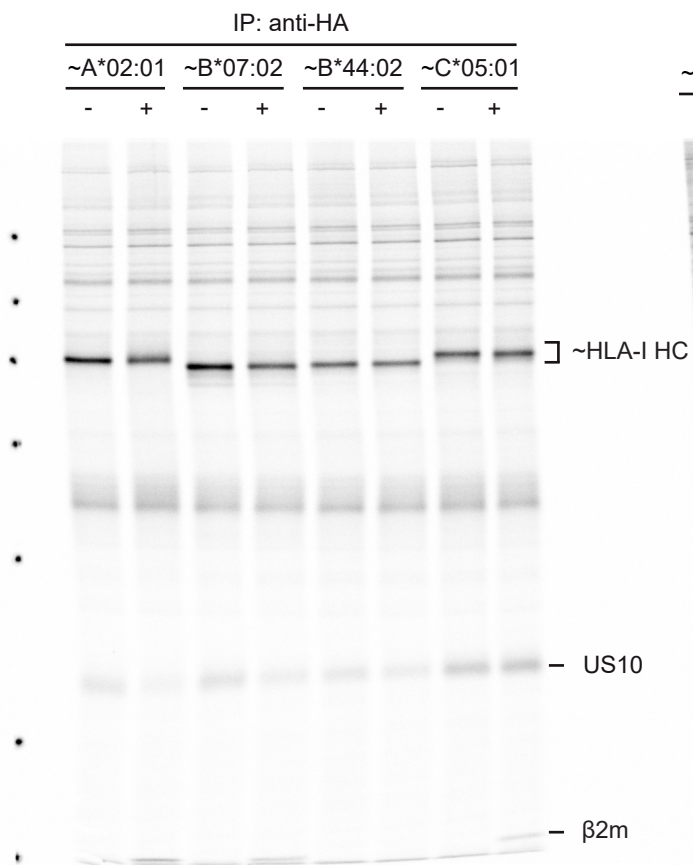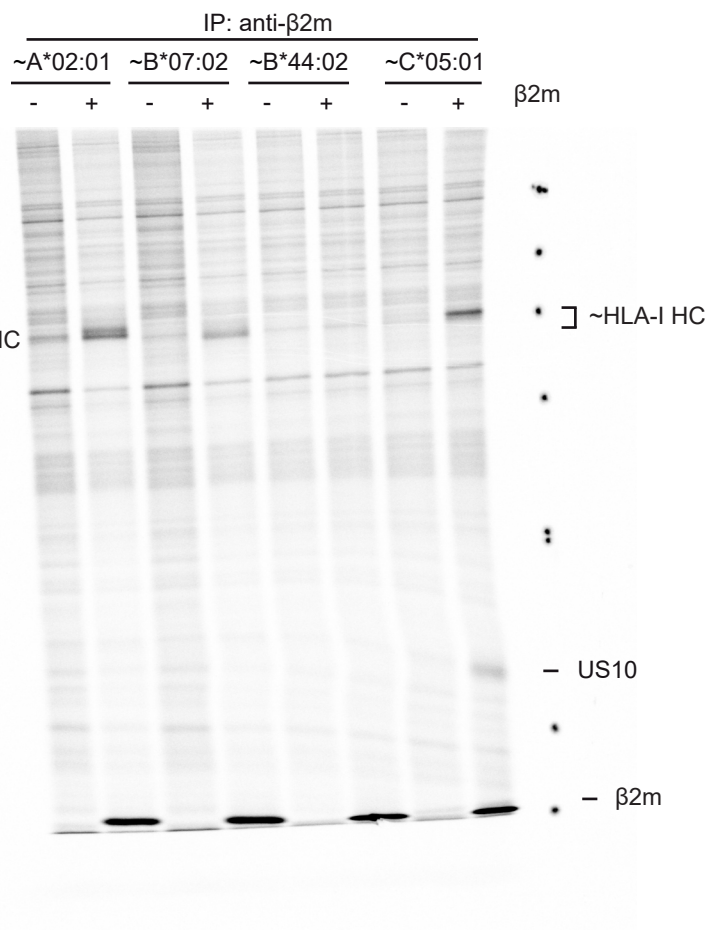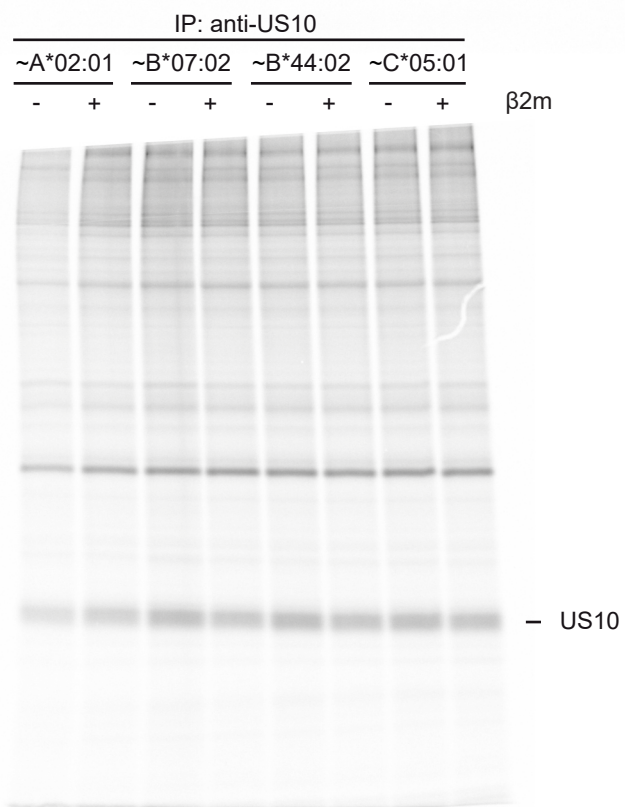

Supplement: Figure 4—source data 4. [file elife-85560-fig4-data4.zip › Figure 4 - Source data 4/Figure_4D_uncropped-1.pdf]

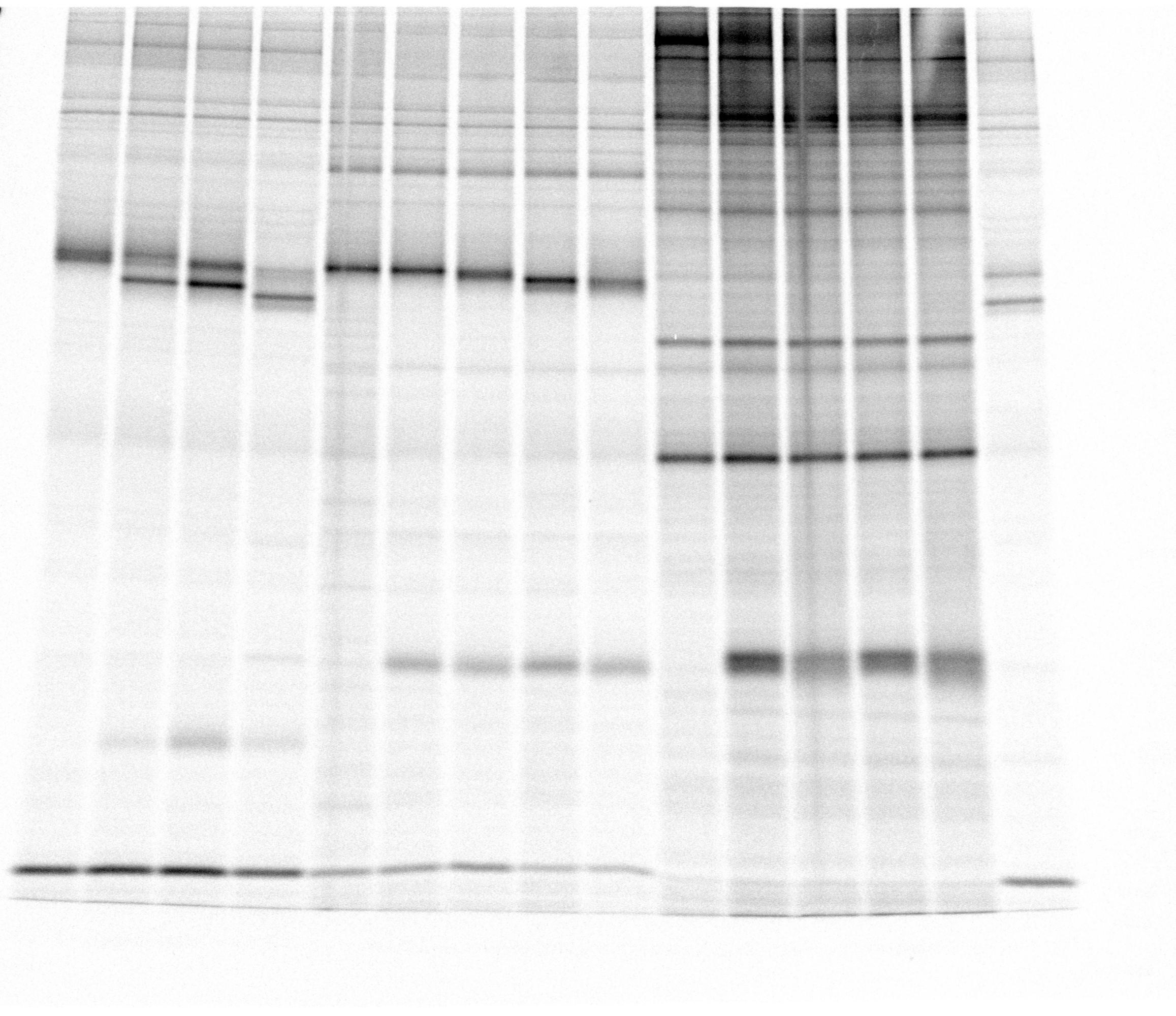

Supplement: Figure 4—source data 5. [file elife-85560-fig4-data5.zip › Figure 4 - Source data 5/Figure 4 - Source data 5_raw data.tif]

IP: W6/32

|   |   |   |
|---|---|---|
| - | + | + |
| - | - | + |
| - | + | + |

IP: anti-US10

|   |   |   |
|---|---|---|
| - | + | + |
| - | - | + |
| - | + | + |

US10  
BFA  
EndoH

- ~A\*02:01 HC +CHO  
- ~A\*02:01 HC -CHO

- US10 +CHO

- US10 -CHO

-  $\beta$ 2m

1 2 3

1 2 3

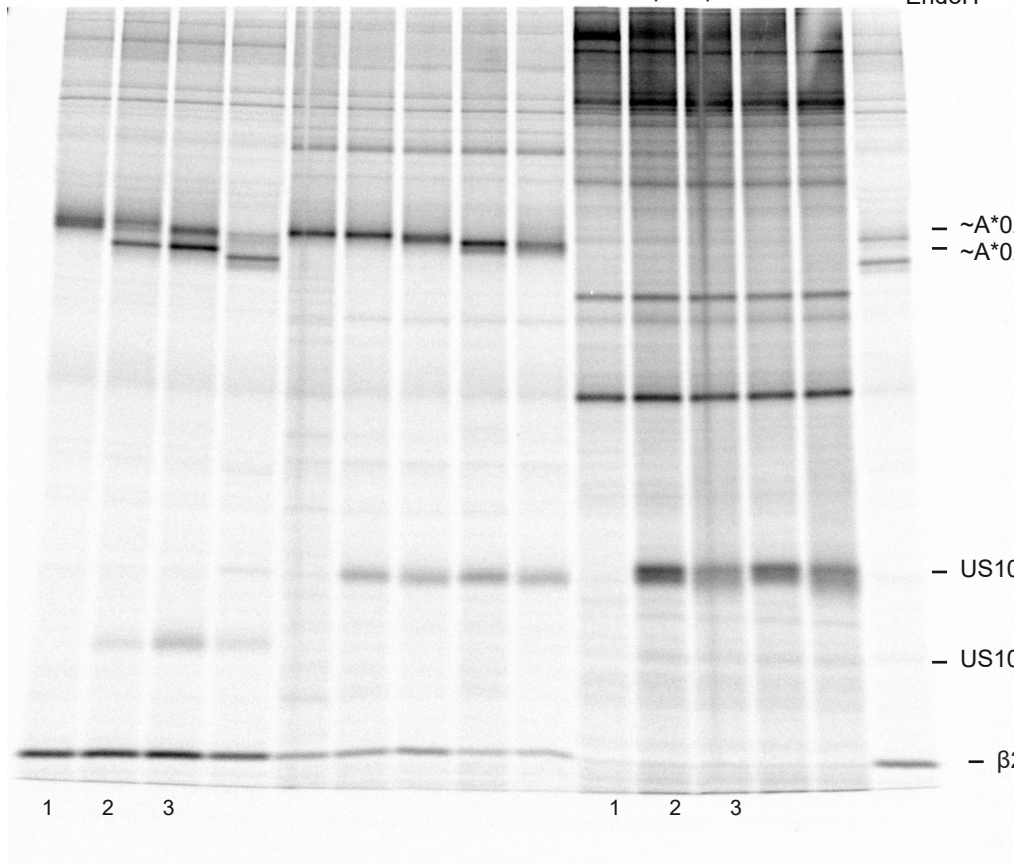

Supplement: Figure 4—source data 5. [file elife-85560-fig4-data5.zip › Figure 4 - Source data 5/Figure_4F_uncropped.pdf]

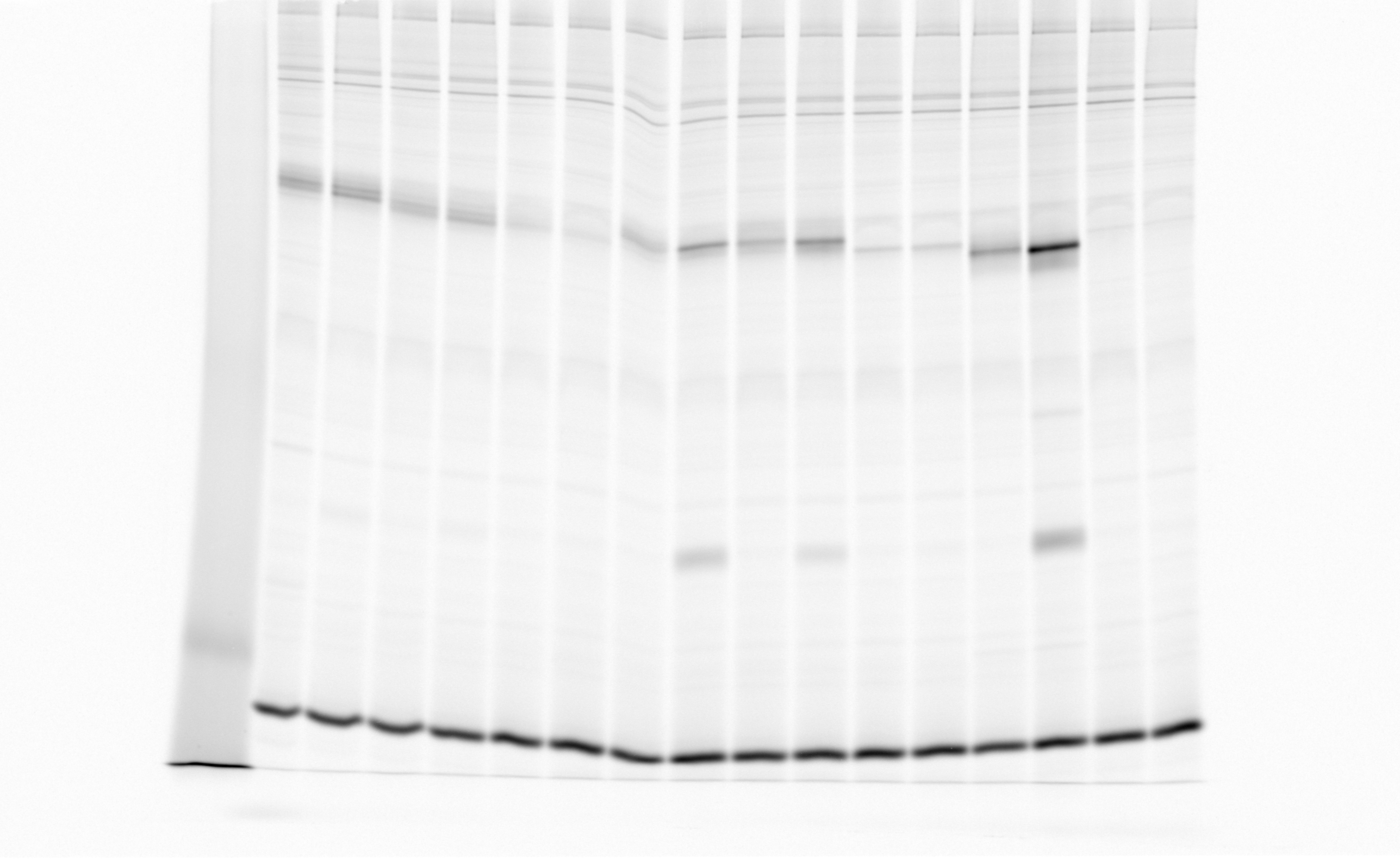

Supplement: Figure 4—figure supplement 1—source data 1. [file elife-85560-fig4-figsupp1-data1.zip › Figure 4 - Figure supplement 1 - Source data 1/Figure 4 - Figure supplement 1_source data 1_anti-b2m_raw data.tif]

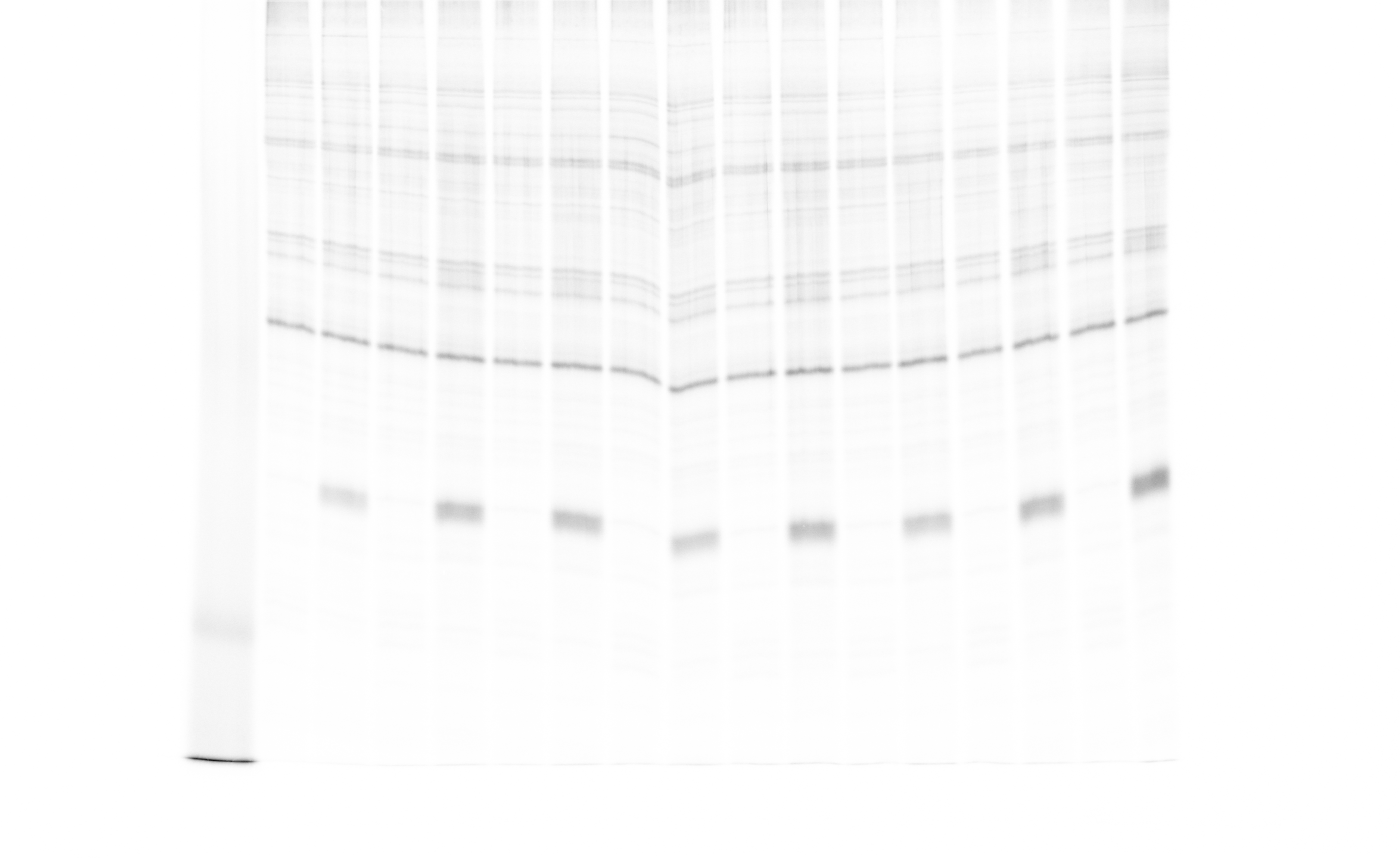

Supplement: Figure 4—figure supplement 1—source data 1. [file elife-85560-fig4-figsupp1-data1.zip › Figure 4 - Figure supplement 1 - Source data 1/Figure 4 - Figure supplement 1_source data 1_anti-US10_raw data.tif]

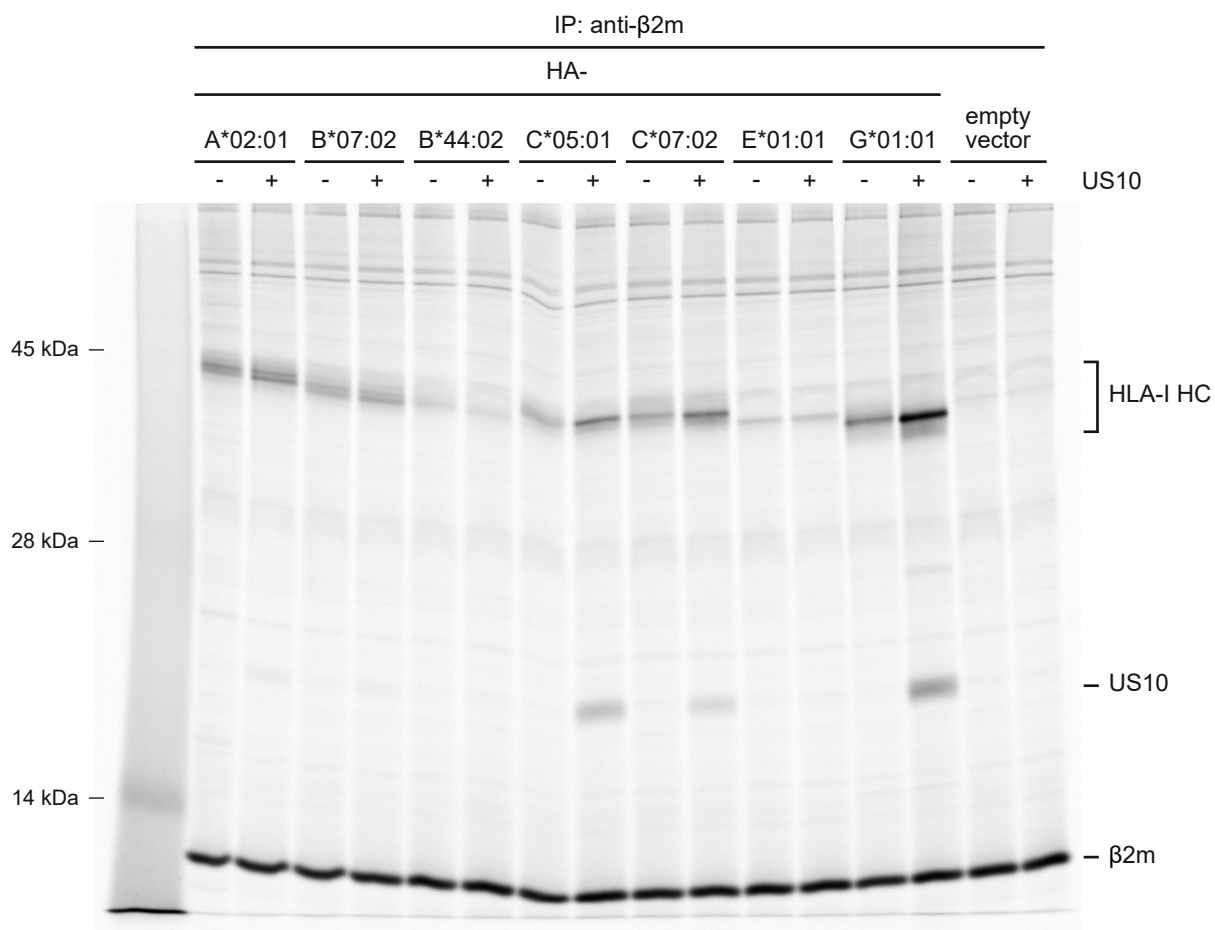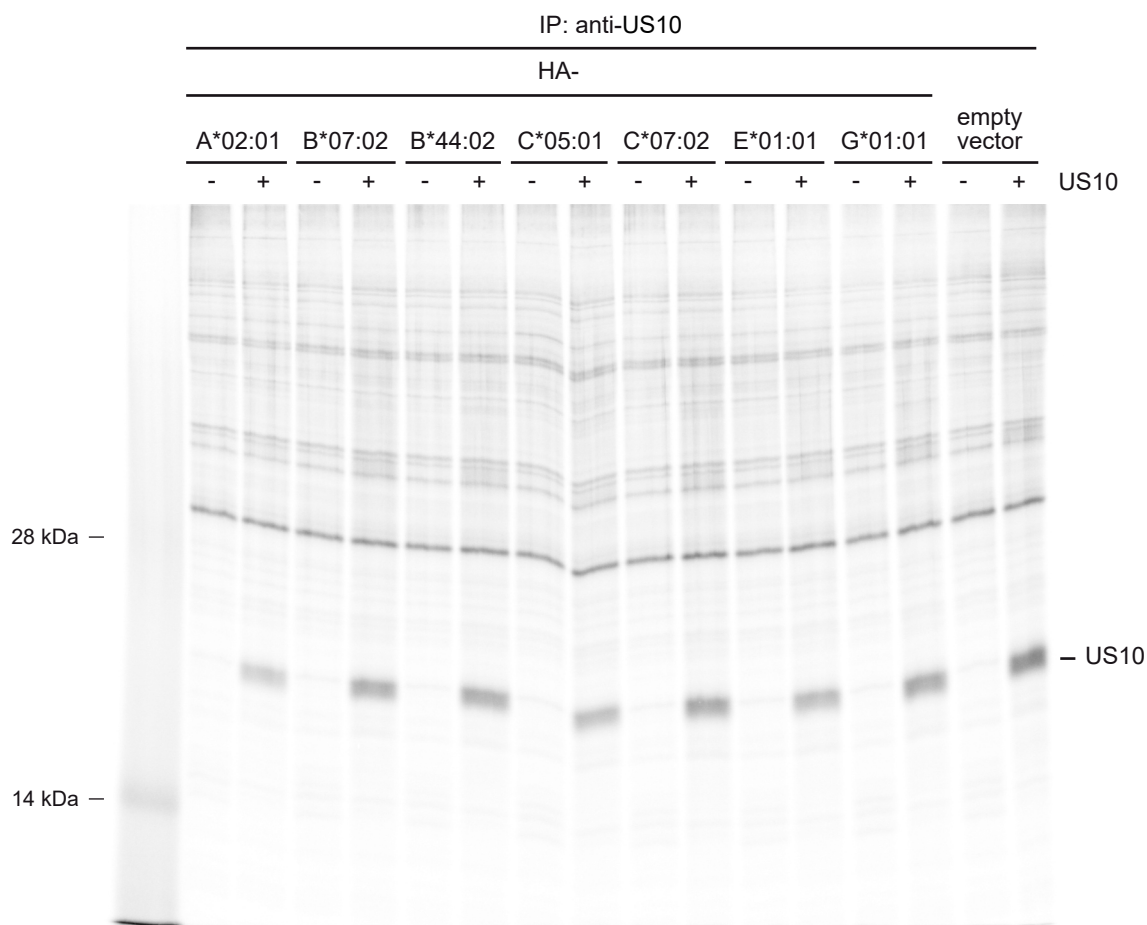

Suppl. Figure 3C: uncropped gels

Supplement: Figure 4—figure supplement 1—source data 1. [file elife-85560-fig4-figsupp1-data1.zip › Figure 4 - Figure supplement 1 - Source data 1/Suppl_figure_3A_uncropped.pdf]

IP: W6/32

+

+

+

IP: anti-US10

+

-

+

IP: W6/32

+

+

+

US10

BFA

EndoH

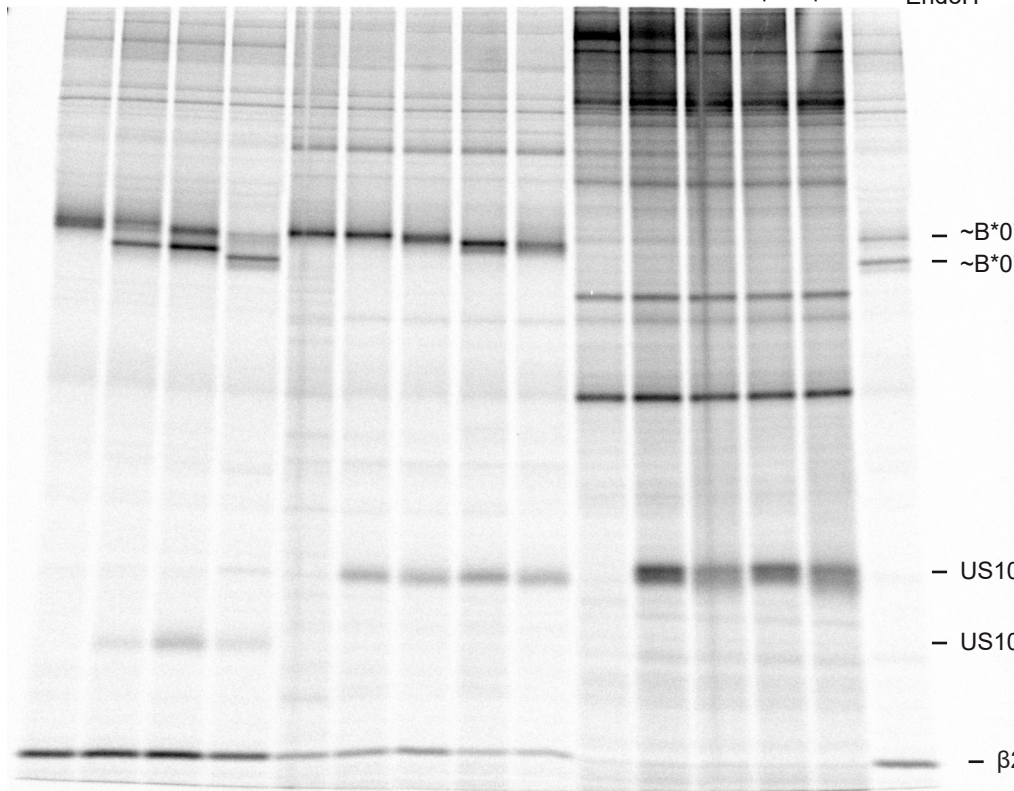

- ~B\*07:02 HC +CHO

- ~B\*07:02 HC -CHO

- US10 +CHO

- US10 -CHO

- β2m

Supplement: Figure 4—figure supplement 1—source data 2. [file elife-85560-fig4-figsupp1-data2.zip › Figure 4 - Figure supplement 1 - Source data 2/Suppl_Figure_3E_uncropped.pdf]

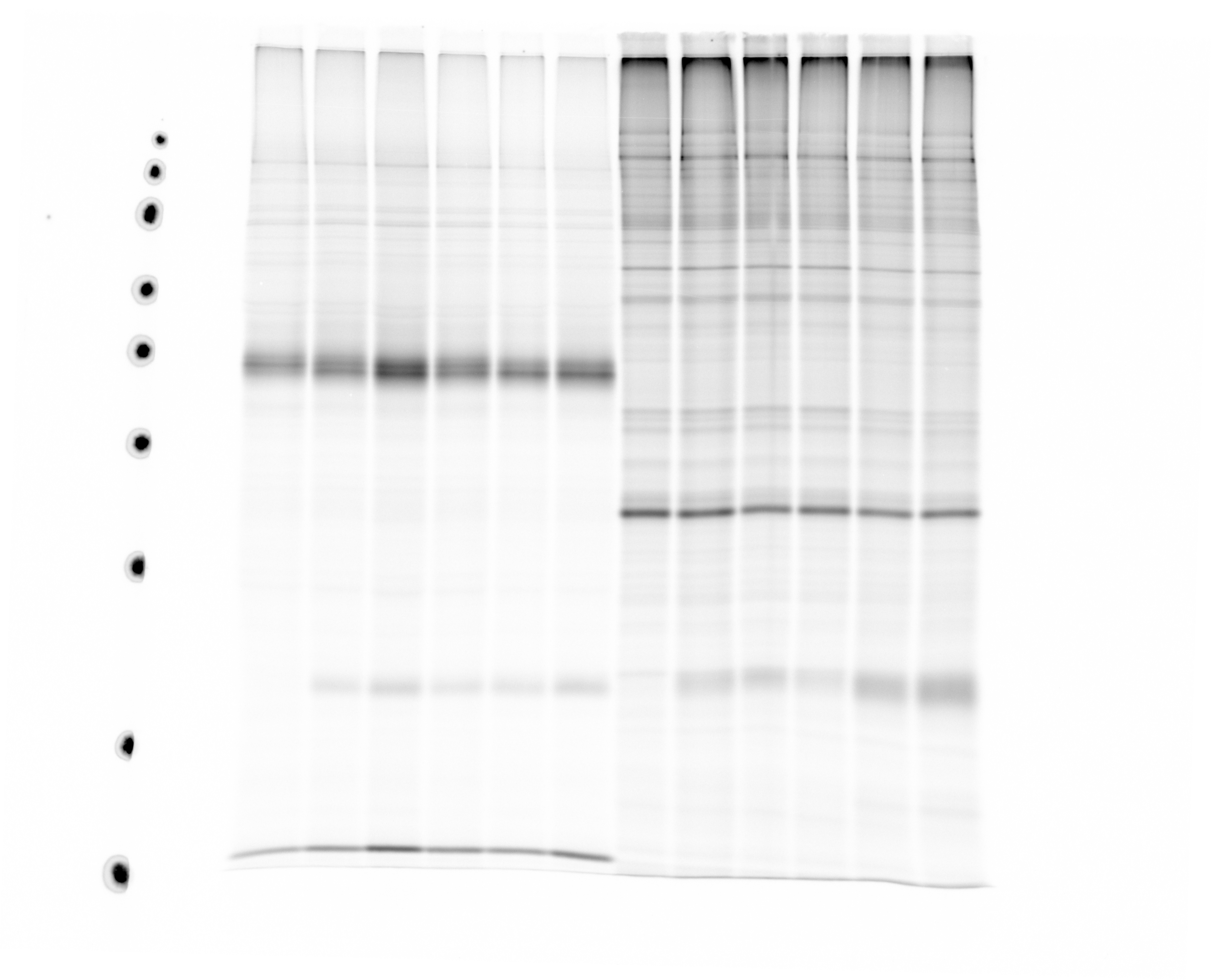

Supplement: Figure 5—figure supplement 1—source data 1. [file elife-85560-fig5-figsupp1-data1.zip › Figure 5 - Figure supplement 1 - Source data 1/Figure 5 - Figure supplement 1 - Source data 1_raw data.tif]

IP: W6/32

IP: anti-US10

wild-type

cell pool

#3

#4

#5

#10

wild-type

cell pool

#3

#4

#5

#10

HLA-I HC

US10

$\beta$ 2m

1

2

3

4

5

6

7

8

9

10

11

12

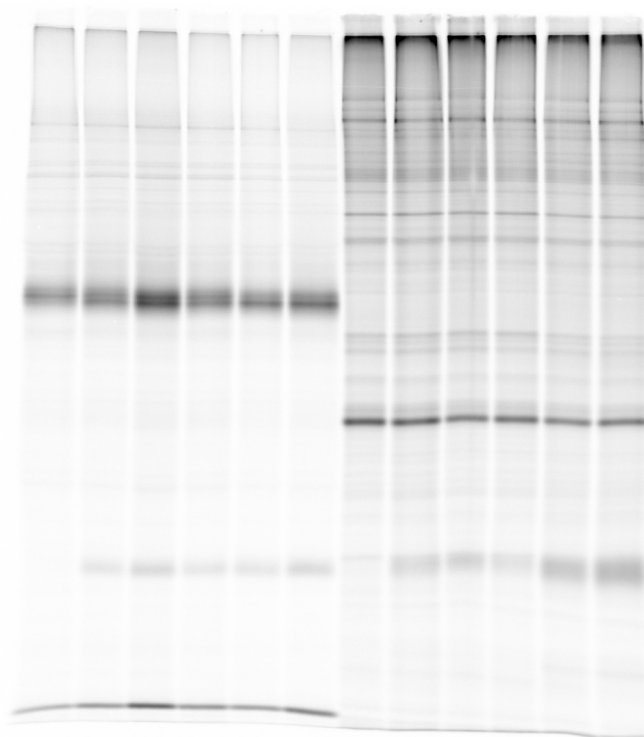

Supplement: Figure 5—figure supplement 1—source data 1. [file elife-85560-fig5-figsupp1-data1.zip › Figure 5 - Figure supplement 1 - Source data 1/Suppl_Figure_4B_uncropped.pdf]

IP: anti-US10

wild-  
type

#5

+

+

-

dox

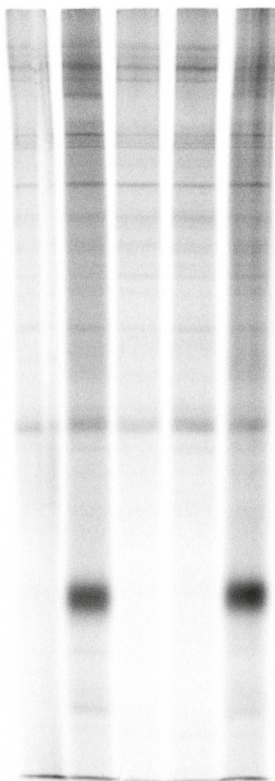

- US10

1

2

3

Supplement: Figure 5—figure supplement 1—source data 2. [file elife-85560-fig5-figsupp1-data2.zip › Figure 5 - Figure supplement 1 - Source data 2/Suppl_Figure_4C_uncropped.pdf]

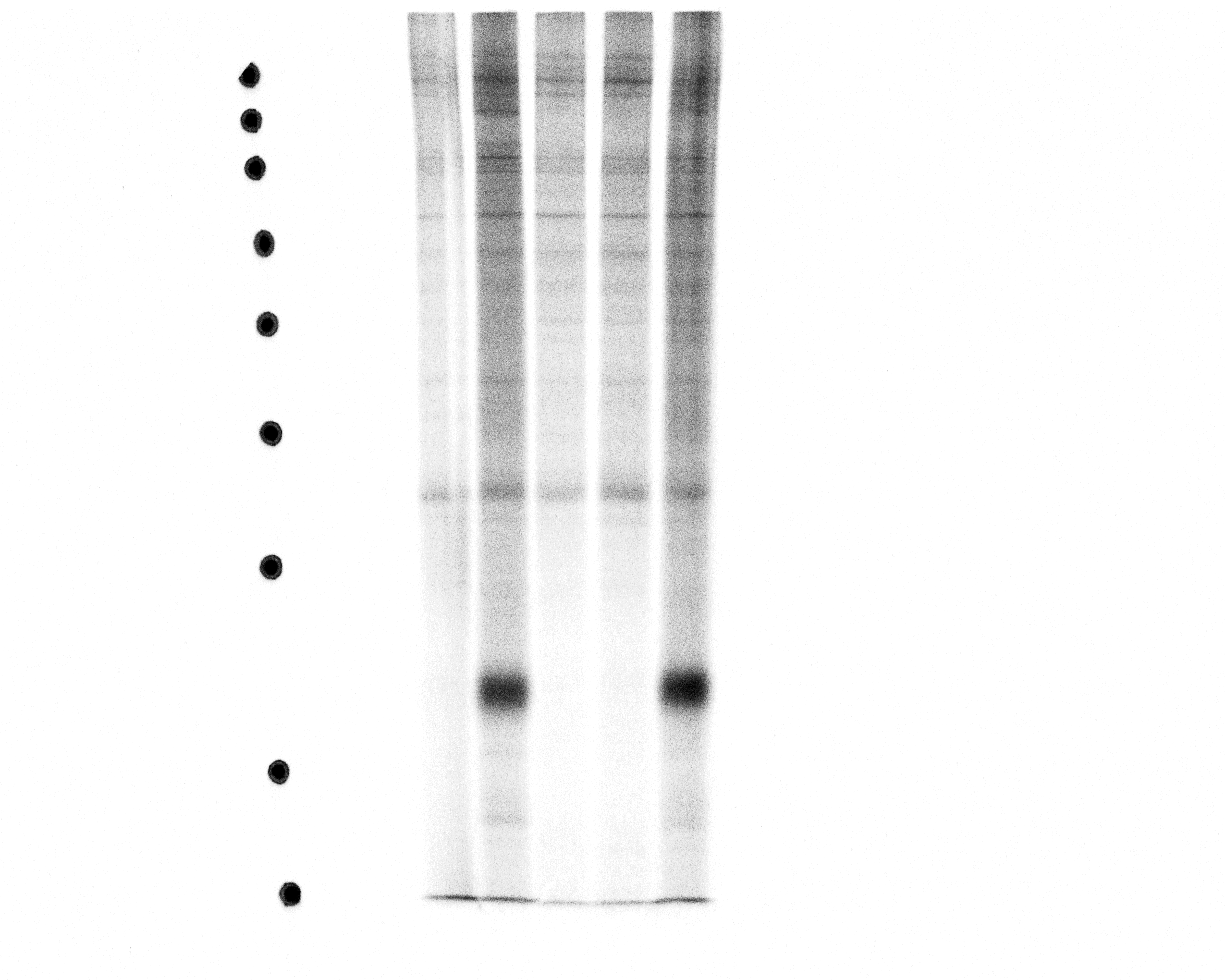

Supplement: Figure 5—figure supplement 1—source data 2. [file elife-85560-fig5-figsupp1-data2.zip › Figure 5 - Figure supplement 1 - Source data 2/Figure 5 - Figure supplement 1 - Source data 2_raw data.tif]

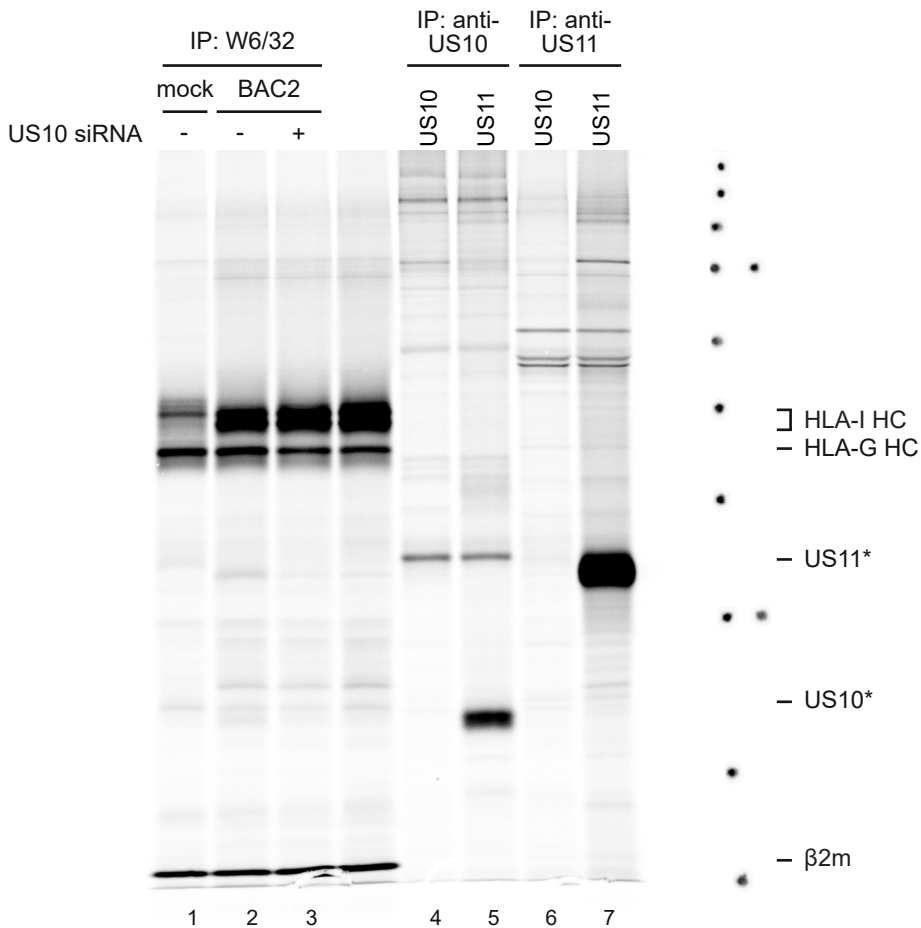

Supplement: Figure 6—source data 1. [file elife-85560-fig6-data1.zip › Figure 6 - Source data 1/Figure_6B_uncropped.pdf]

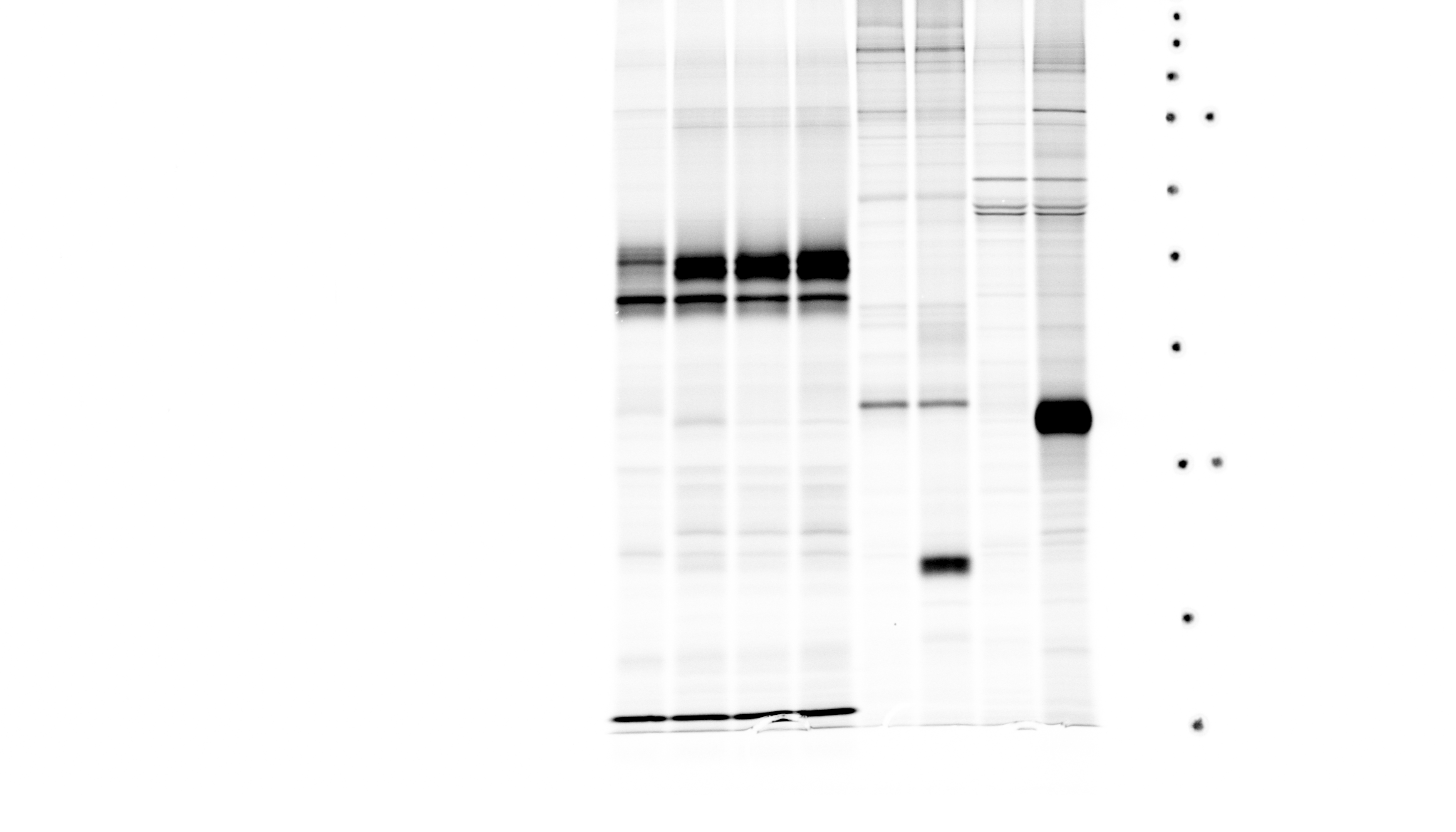

Supplement: Figure 6—source data 1. [file elife-85560-fig6-data1.zip › Figure 6 - Source data 1/Figure 6 - Source data 1_raw data.tif]

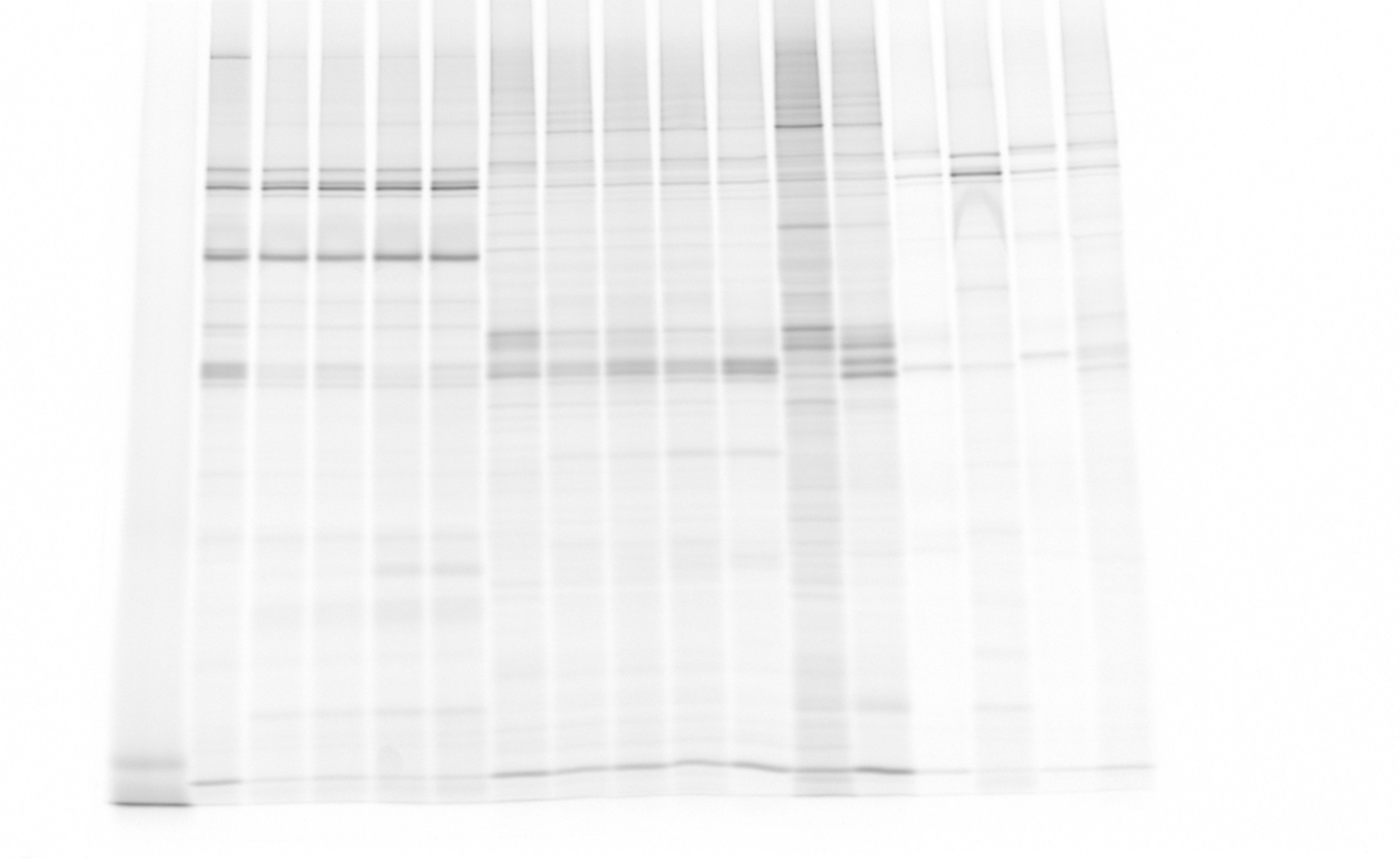

Supplement: Figure 6—source data 2. [file elife-85560-fig6-data2.zip › Figure 6 - Source data 2/Figure 6 - Source data 2_raw data.tif]

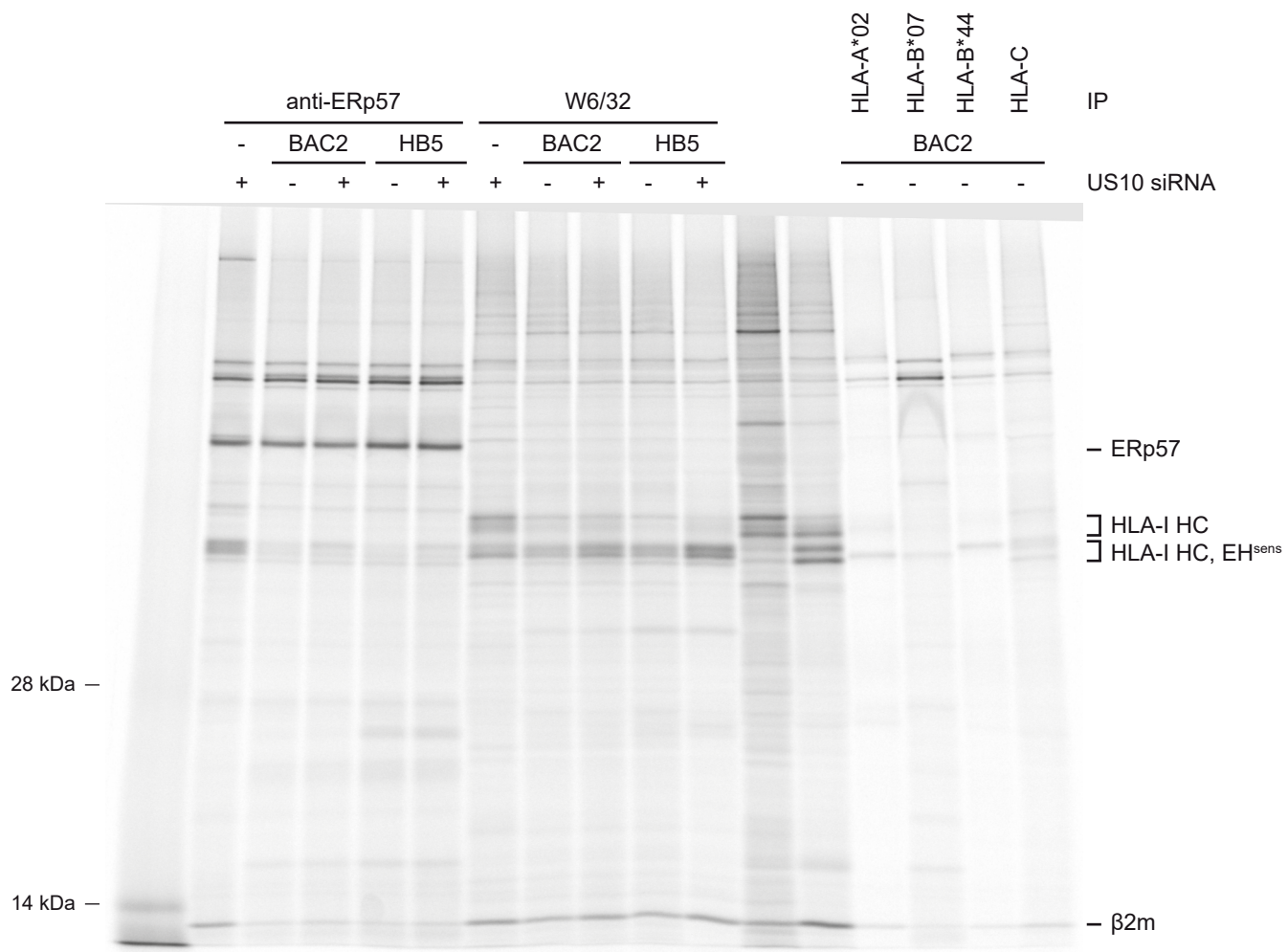

Figure 7CD: uncropped gel

Supplement: Figure 6—source data 2. [file elife-85560-fig6-data2.zip › Figure 6 - Source data 2/Figure_6CD_uncropped.pdf]

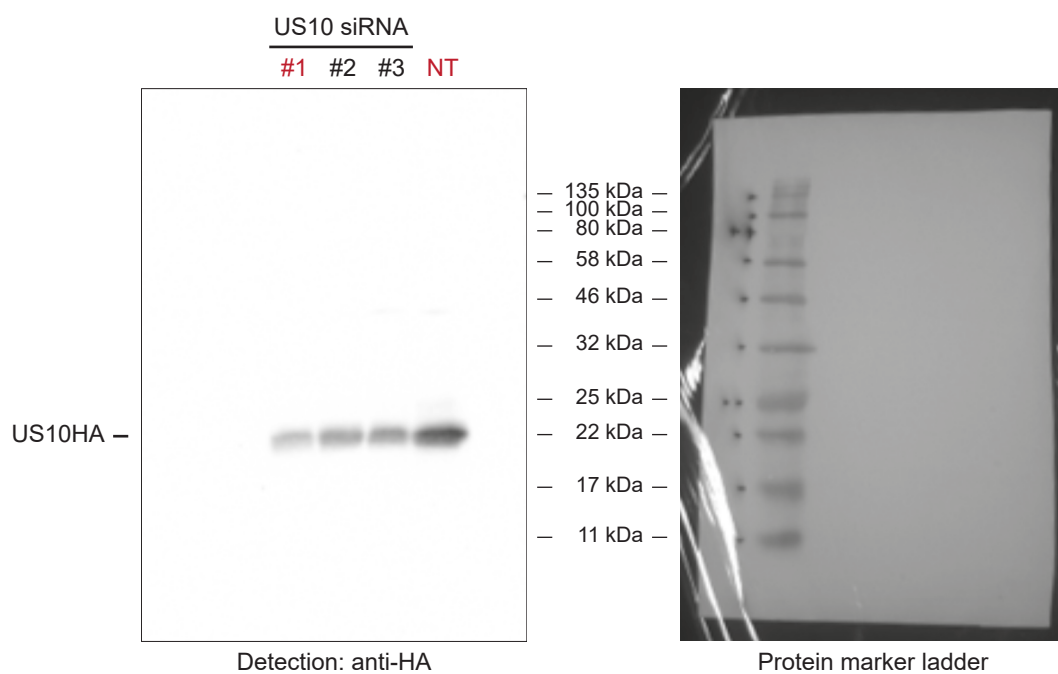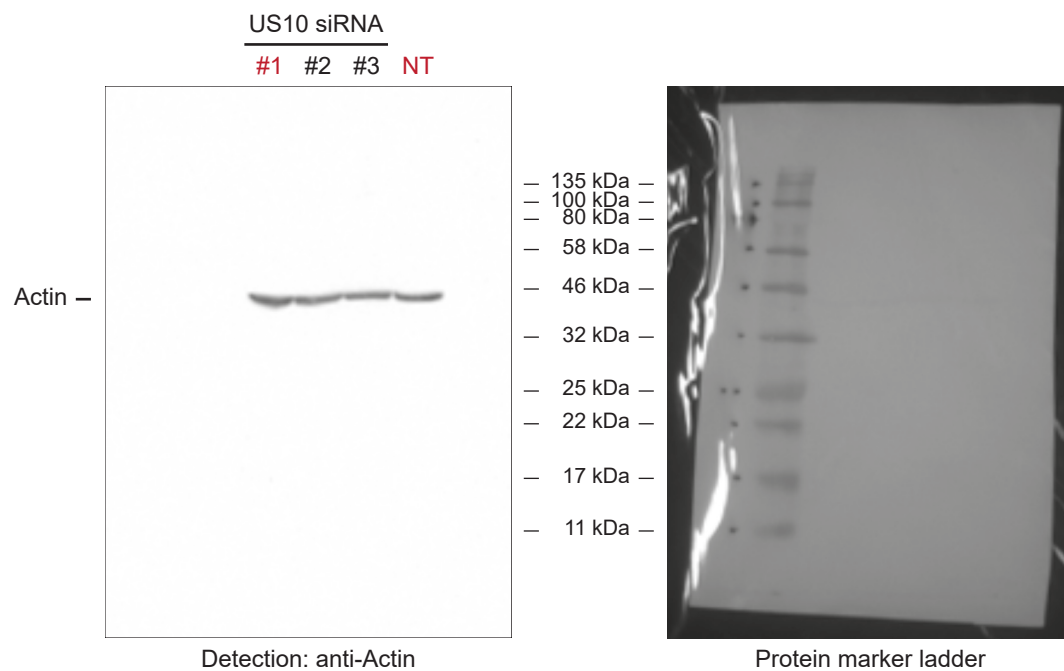

Suppl. Figure 5C: uncropped blots

Supplement: Figure 6—figure supplement 1—source data 1. [file elife-85560-fig6-figsupp1-data1.zip › Figure 6 - Figure supplement 2 - Source data 1/Suppl_figure_5D_uncropped.pdf]

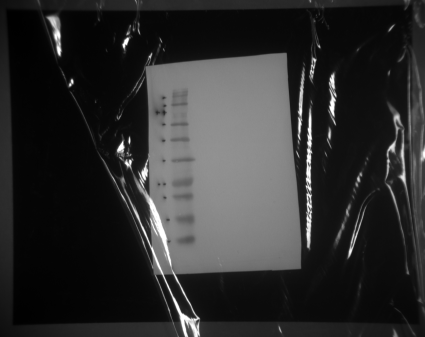

Supplement: Figure 6—figure supplement 1—source data 1. [file elife-85560-fig6-figsupp1-data1.zip › Figure 6 - Figure supplement 2 - Source data 1/Figure 6 - Figure supplement 2 - Source data 1_anti-HA_ladder_raw data.tif]

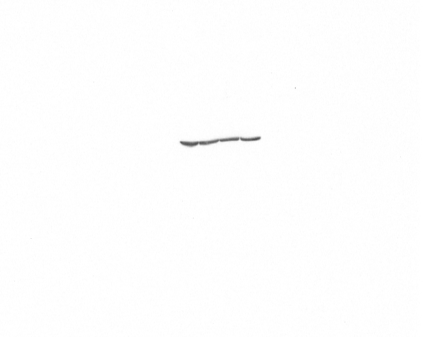

Supplement: Figure 6—figure supplement 1—source data 1. [file elife-85560-fig6-figsupp1-data1.zip › Figure 6 - Figure supplement 2 - Source data 1/Figure 6 - Figure supplement 2 - Source data 1_anti-Actin_raw data.tif]

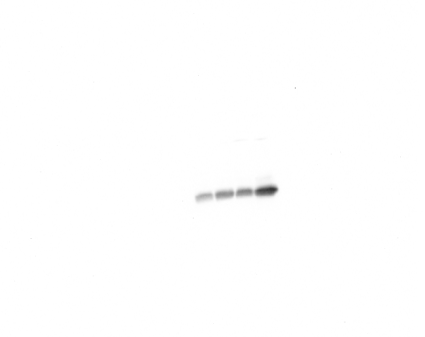

Supplement: Figure 6—figure supplement 1—source data 1. [file elife-85560-fig6-figsupp1-data1.zip › Figure 6 - Figure supplement 2 - Source data 1/Figure 6 - Figure supplement 2 - Source data 1_anti-HA_raw data.tif]

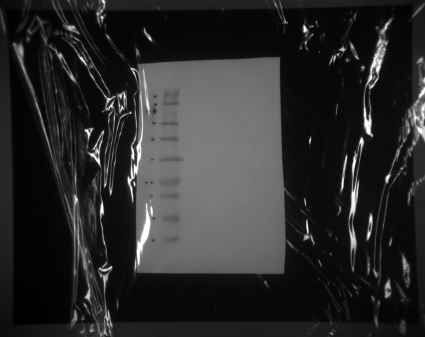

Supplement: Figure 6—figure supplement 1—source data 1. [file elife-85560-fig6-figsupp1-data1.zip › Figure 6 - Figure supplement 2 - Source data 1/Figure 6 - Figure supplement 2 - Source data 1_anti-Actin_ladder_raw data.tif]

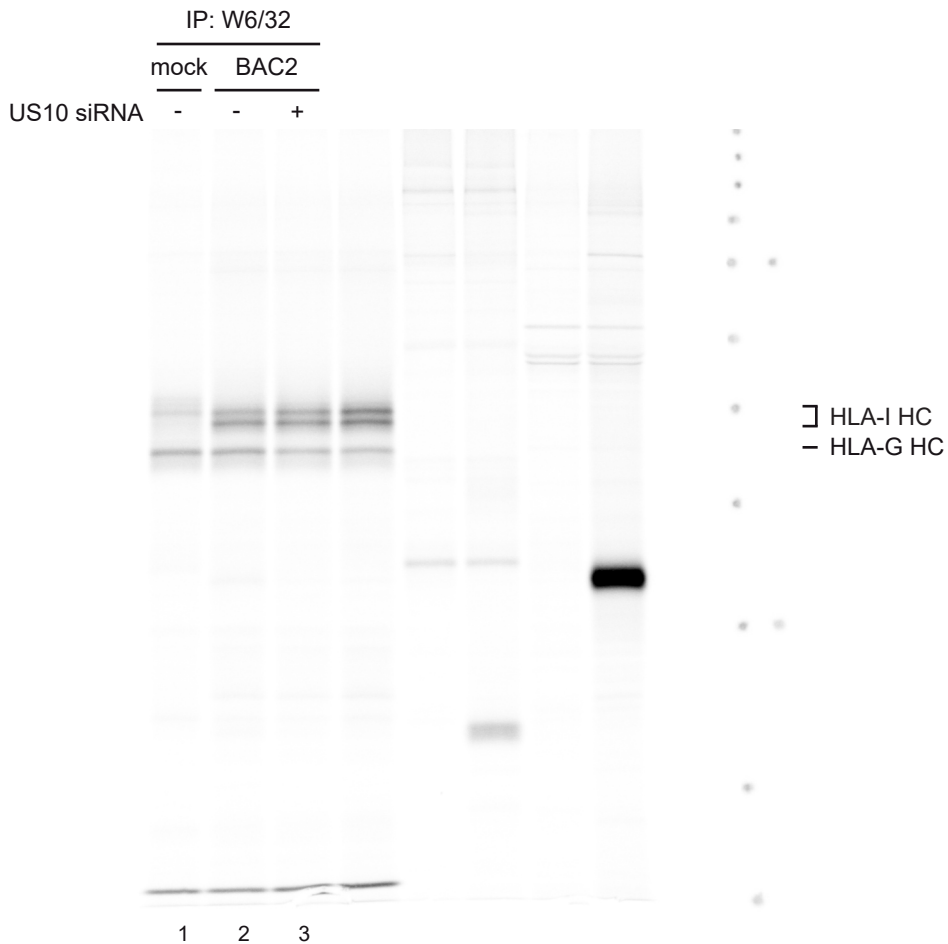

Supplement: Figure 6—figure supplement 2—source data 2. [file elife-85560-fig6-figsupp2-data2.zip › Figure 6 - Figure supplement 2 - Source data 2/Suppl_Figure_5G_uncropped.pdf]

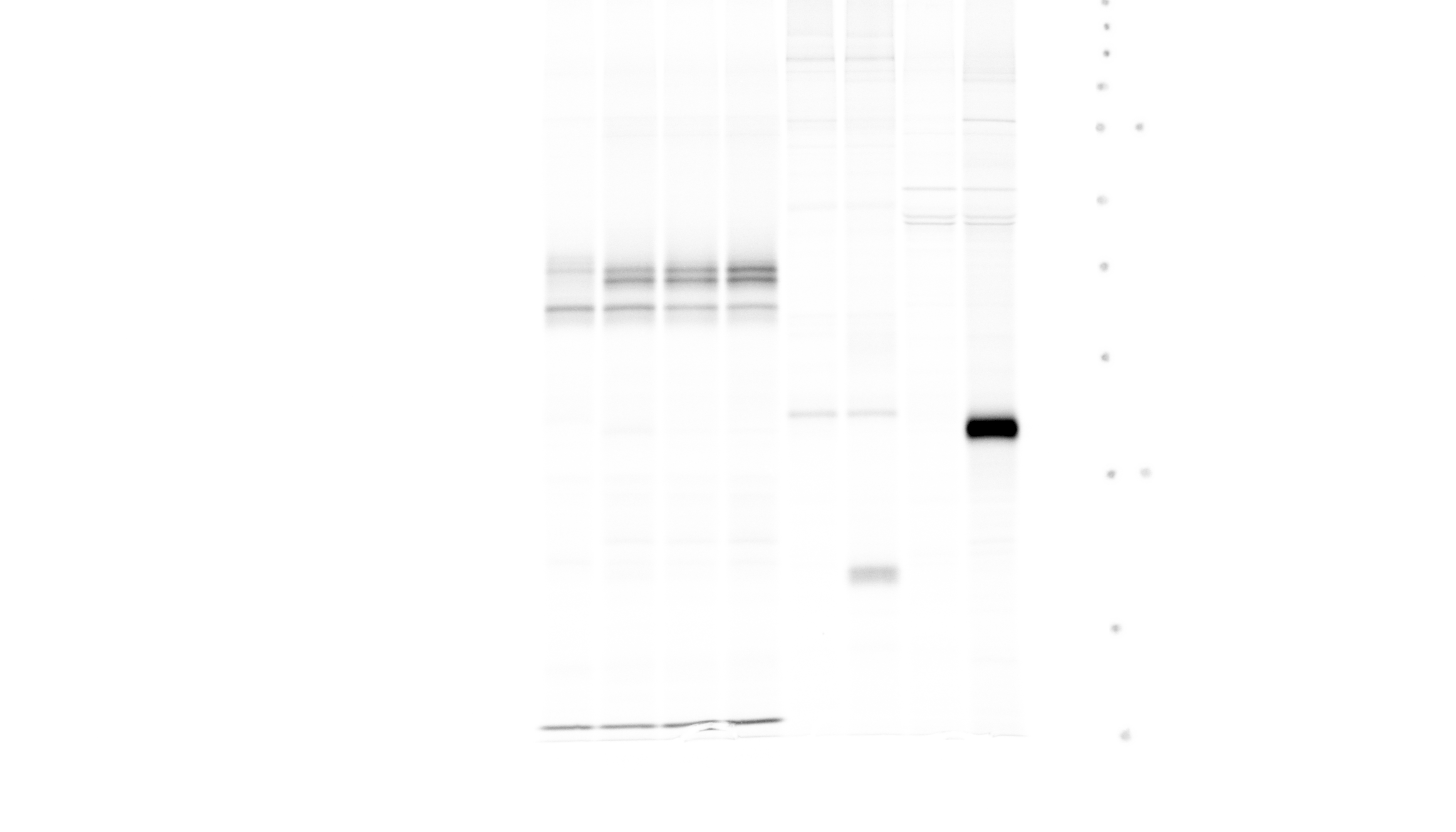

Supplement: Figure 6—figure supplement 2—source data 2. [file elife-85560-fig6-figsupp2-data2.zip › Figure 6 - Figure supplement 2 - Source data 2/Figure 6 - Figure supplement 2 - Source data 2.tif]
