# Supplementary material for: Multimodal HLA-I genotype regulation by human cytomegalovirus US10 and resulting surface patterning
Source: eLife. 2024 Jun 20;13:e85560. doi: 10.7554/eLife.85560 (PMC11189632; doi:10.7554/eLife.85560)
Supplement: Figure 3—figure supplement 1—source data 1. [file elife-85560-fig3-figsupp1-data1.zip › Figure 3 - Figure supplement 1 - Source data 1/Suppl_figure_2A_uncropped.pdf]

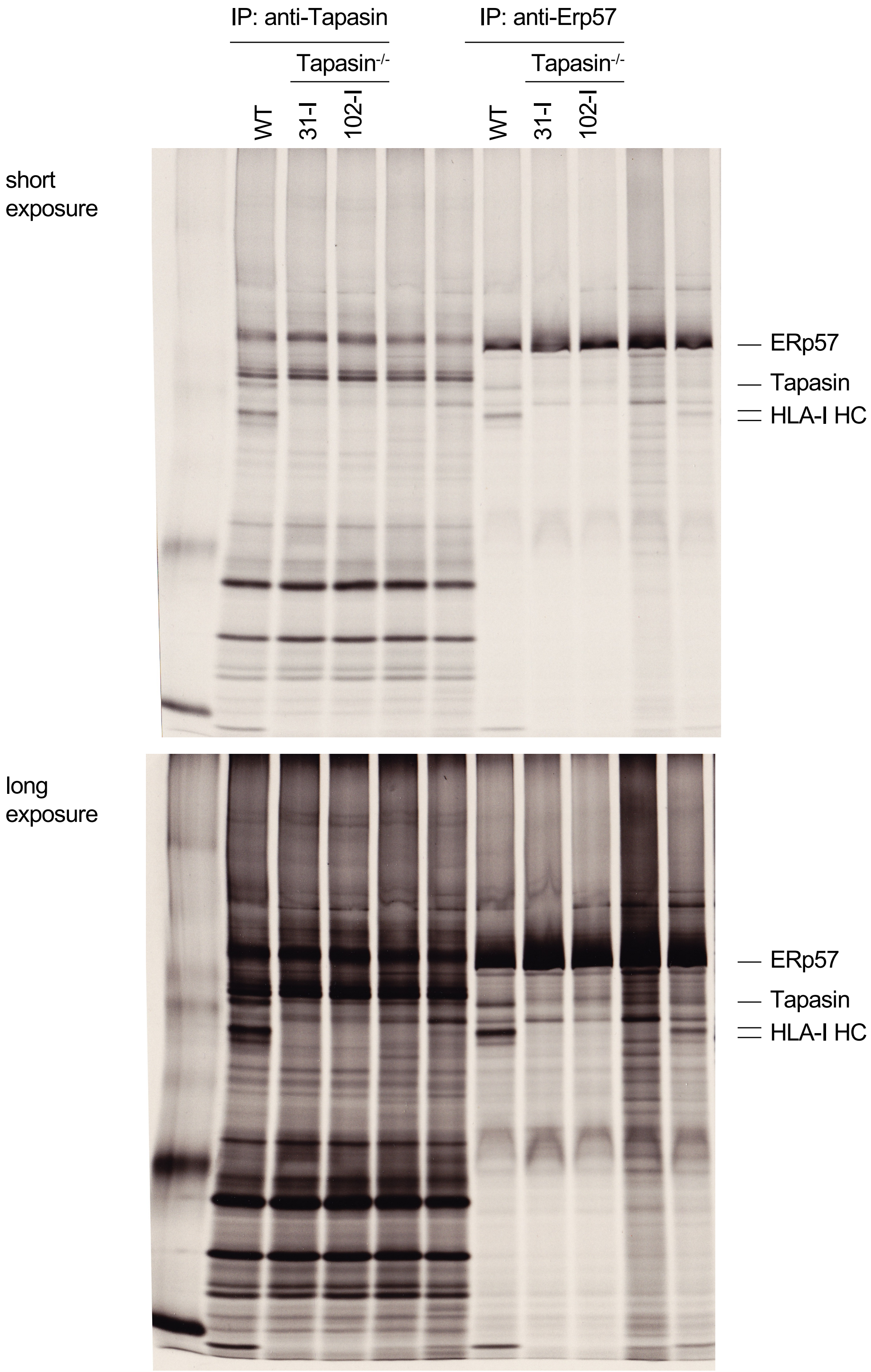
IP: anti-Tapasin
Tapasin-/-
WT
31-I
102-I
IP: anti-Erp57
Tapasin-/-
WT
31-I
102-I
short exposure
ERp57
Tapasin
HLA-I HC
long exposure
ERp57
Tapasin
HLA-I HC
